# Mechanics of ultrasound elastography

**DOI:** 10.1098/rspa.2016.0841

**Published:** 2017-03-01

**Authors:** Guo-Yang Li, Yanping Cao

**Affiliations:** Department of Engineering Mechanics, Institute of Biomechanics and Medical Engineering, AML, Tsinghua University, Beijing 100084, People's Republic of China

**Keywords:** ultrasound elastography, shear wave, soft tissues

## Abstract

Ultrasound elastography enables *in vivo* measurement of the mechanical properties of living soft tissues in a non-destructive and non-invasive manner and has attracted considerable interest for clinical use in recent years. Continuum mechanics plays an essential role in understanding and improving ultrasound-based elastography methods and is the main focus of this review. In particular, the mechanics theories involved in both static and dynamic elastography methods are surveyed. They may help understand the challenges in and opportunities for the practical applications of various ultrasound elastography methods to characterize the linear elastic, viscoelastic, anisotropic elastic and hyperelastic properties of both bulk and thin-walled soft materials, especially the *in vivo* characterization of biological soft tissues.

## 1. Introduction

**Figure 1.**
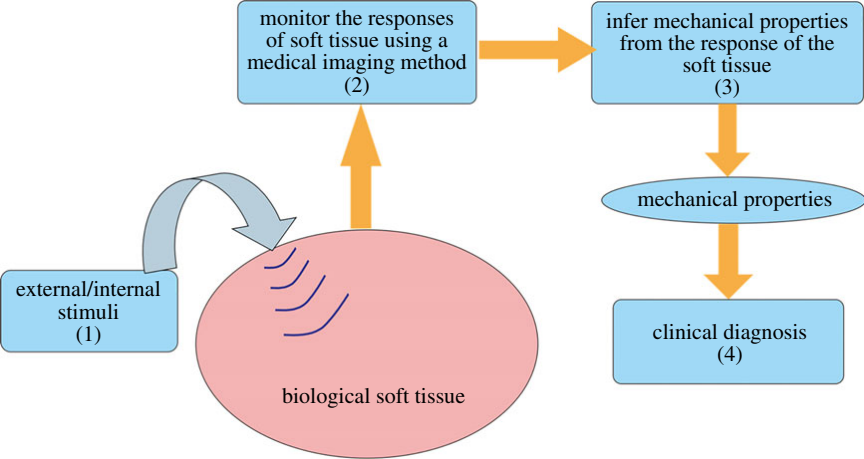
An illustration of the key steps involved in elastography [8]. (Online version in colour.)

The elastography method, which was proposed in the 1990s, enables probing the elastic properties of living soft tissues and has found wide medical applications in the past two decades [1–7]. The key steps involved in an elastography method can be summarized as in figure 1 [8]. (1) An external or internal stimulus is imposed onto a target soft tissue. (2) The responses of the soft tissue, including its static and/or dynamic deformation behaviours are monitored using a medical imaging technique, such as ultrasound or nuclear magnetic resonance imaging (MRI) methods. (3) The mechanical properties of the soft tissue can then be inferred from the measured responses based on inverse analysis. (4) It has been recognized that many diseases, such as cancer [9,10], liver fibrosis [11,12], cardiovascular diseases [13] and thyroid nodules [14], are accompanied by variations in the tissue mechanical properties; therefore, *in vivo* and quantitative measurements of the elastic properties of soft tissues via elastography methods provide valuable information for the diagnosis and therapy of these diseases.

The vast studies published in the literature regarding the development and practical applications of elastography methods may be classified by considering the four key steps in figure 1. In step (1), different stimuli can be adopted to deform a soft tissue. In the literature, static loads [15], external vibrators [16,17] and acoustic radiation forces (ARFs) [18–21] have been applied to generate diverse responses in a soft tissue, leading to different static and dynamic elastography methods. Accurately tracking the mechanical responses of target soft tissues generated by various stimuli (step (2)) is a key step in an elastography method. To this end, different medical imaging methods have been used, giving rise to ultrasound elastography, magnetic resonance elastography, optical elastography and so on [18,22–24]. Also driven by this need, some dedicated imaging techniques have been introduced. For instance, besides the measurement of axial displacements, techniques based on the ultrasound imaging have been presented to obtain the lateral displacements and strains [25]. Another example is that taking advantage of ultrafast ultrasound imaging techniques (the frame rate can be up to 6000 Hz or even higher), the method to image two-dimensional motion vectors has been developed [24]. With the known responses of soft tissues under given stimuli tracked with various medical imaging methods, it is possible to infer the mechanical properties of soft tissues (step (3) in figure 1), which has received considerable attention from different disciplines. Besides linear elastic parameters, it has been demonstrated that hyperelastic [8,26–28], viscoelastic [29–32] and anisotropic elastic [33–36] parameters of soft tissues may be inferred using different inverse methods reported in recent years. The mechanical properties of living soft tissues inferred from their responses to imposed stimuli may provide valuable information for the diagnosis and therapy of some diseases (step (4)). This step is the main interest of clinicians who use elastography, and most publications from clinical research focus on this aspect. Over the past years, numerous valuable clinical data have been reported in the literature, which indeed help identify the extent to which elastography methods are useful in clinics [10,12,37].

This review focuses on ultrasound elastography for which quite a few review papers [1–7] and guidelines for its clinical use [38,39] have been published. It can be seen from figure 1 that continuum mechanics plays an essential role in both steps (1) and (3). In particular, the questions of how to understand the responses of living soft tissues to various external/internal stimuli in ultrasound elastography and how to establish robust inverse approaches to infer different material parameters of soft tissues have come under the spotlight of the mechanics and applied mathematics research communities. Bearing this issue in mind, distinct from previous review papers, this review focuses on the mechanics principles underpinning elastography methods to highlight the limitations, challenges and opportunities of these methods from the viewpoint of continuum mechanics. To this end, we divide the commonly used ultrasound elastography methods into three categories based on the loads or stimuli imposed on the soft tissue and its responses: static elastography, dynamic elastography with harmonic stimuli (DEHS) and dynamic elastography with transient stimuli (DETS). From the viewpoint of continuum mechanics, the governing equations and boundary conditions (BCs) characterizing the responses of soft tissues involved in these three types of methods differ.

This review paper is organized as follows. In §2, a brief introduction to the commonly used ultrasound elastography methods and their applications is presented. Section 3 describes the mechanics theories involved in elastography methods. In §4, particular attention is given to some limitations of the current data analysis methods and future prospects for developing novel inverse analysis methods within the framework of continuum mechanics. Section 5 provides the concluding remarks.

## 2. Ultrasound-based elastography methods

Ultrasound imaging is a low-cost, safe and mobile imaging modality that can generate real-time images and has found broad applications in clinical radiology. Safety is one of its major strengths; indeed, this technique does not involve ionizing radiations. Ultrasound-based elastography methods use ultrasound imaging to track the deformation behaviours of soft tissues and further infer the elastic properties of both healthy and diseased soft tissues. Depending on the features of the stimuli used to deform the soft tissue, the ultrasound-based elastography methods can be divided into three categories: static elastography, DEHS and DETS. This section gives an overview of different ultrasound-based elastography techniques and their applications for the mechanical characterization of soft tissues and diagnosis of some diseases.

### (a) Static elastography methods

**Figure 2.**
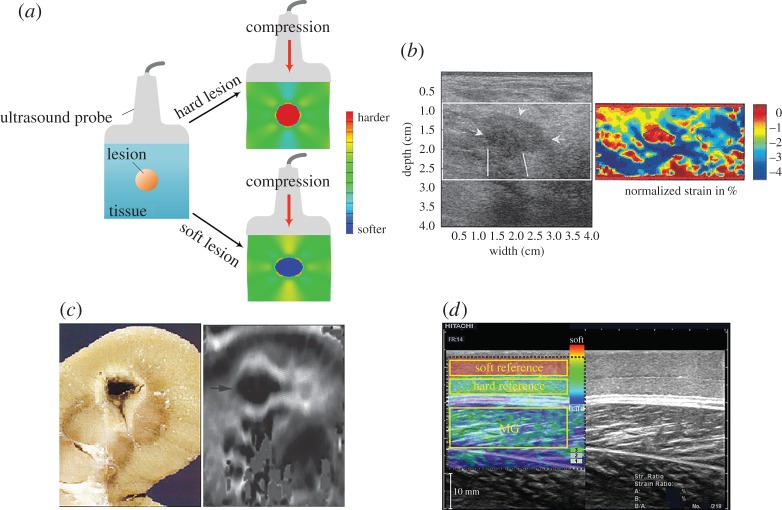
(*a*) Illustration of the basic principle underlying static elastography. (*b*) Detection of a breast lesion, which appears as the low-strain region in the image. Compared with the B-mode image, the elastography method may provide more accurate information with respect to both the size and the position of a lesion [45]. (*c*) Application of static elastography to image a thermal lesion induced by HIFU, revealing that this technique may be useful to guide HIFU treatment [46]. (*d*) Measurement of the elastic properties of skeletal muscles using the static elastography method. To quantitatively infer the elastic properties of the muscle, two soft layers with known mechanical properties are used [47]. Reprinted from references [45], [46], and [47] with permission. (Online version in colour.)

Static elastography, which was proposed in the early 1990s, has been widely used in clinics in the past two decades [2,15,25,40–44]. When using this method, static compression is typically imposed onto a targeted soft tissue (figure 2*a*). The resulting displacement field (mainly the axial displacement in the early use of static elastography) generated by the compressive load can be directly measured via the ultrasound imaging method. The strain field can then be calculated according to the measured displacement. Furthermore, dedicated inverse approaches can be used to extract the elastic properties of the target soft tissues according to the strain field [2,6,41,48]. Briefly, harder tissues have lower strains, whereas softer tissues have higher strains under compression, as shown in figure 2*a*. In principle, it is possible to quantitatively infer the elastic properties of soft tissues using a static elastography method; however, this is challenging because of the complexity of the associated inverse problem, as discussed in detail in §3. Therefore, the static elastography method is usually regarded as a qualitative method that reveals the contrast between hard and soft tissues.

Although the limitations of the static elastography method in quantitatively measuring the mechanical properties of soft tissues have been recognized, this method is simple and easy to realize and allows us at least to qualitatively distinguish between regions with different stiffnesses; therefore, it has been widely used in clinics. For example, static elastography finds important applications in the classification of breast lesions [45,49]. A low-echogenic region emerges in the B-mode image when a lesion exists, as shown in figure 2*b*. Traditionally, such a low-echogenic region (the region indicated by arrows in figure 2*b*) in the B-mode image may be suspected to correspond to a breast lesion. Now with the help of the static elastography method, the position and size of a lesion may be detected more accurately than using the result estimated solely based on the B-mode image [45]. Moreover, the mechanical properties of thermal lesions induced by high-intensity focused ultrasound (HIFU) have been demonstrated to differ from those of the surrounding soft tissues; therefore, the static elastography method can be a useful tool to guide HIFU treatment when the lesions are not too deep [46,50]. The dark region in figure 2*c* denotes the low-strain region (i.e. the location of the thermal lesion). In addition to lesion detection, static elastography can also be used to characterize other soft tissues. For instance, in a recent work, Chino *et al*. [47] used two referenced layers with known elastic moduli to cover the skin and measured the compressive strains in both the referenced layers and the skeletal muscles. Although the anisotropy of the skeletal muscles was ignored in their study, by comparing the strains in the referenced layers and skeletal muscles, the elastic properties of the skeletal muscles were evaluated, as shown in figure 2*d* [47]. Other applications of static elastography in predicting malignancies in thyroid nodules can be found in [14,51].

In the clinical use of static elastography methods, the quality of the examination depends significantly on the experience and technique of the sonographer clinicians. For instance, an appropriate compressive amount should be imposed onto the soft tissue to improve the image quality. The appropriate pre-compression of the soft tissues before the imaging process may help increase the contrast and reduce the decorrelation noise [3,46]. However, determining the amount of pre-compression required is by no means trivial because a small pre-compression may not increase the imaging contrast, whereas a large pre-compression may lead to hardening of the soft tissue. This issue will be discussed in detail in §3.

### (b) Dynamic elastography with harmonic stimuli

**Figure 3.**
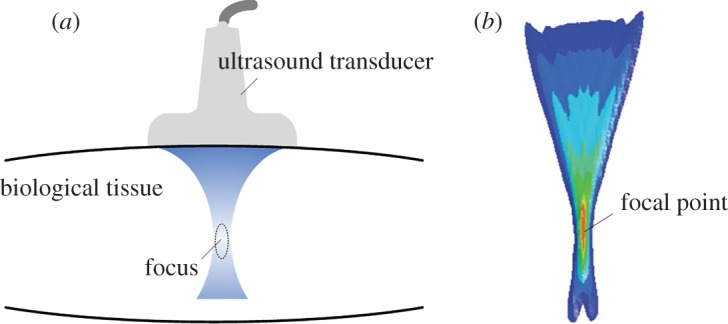
(*a*) Schematic of the ARF in biological soft tissues induced by a focused ultrasound beam. (*b*) The isocontours of the ARF distribution from a focused linear array in a soft medium; here the acoustic attenuation coefficient is 0.7 dB cm^−1^ MHz [52]. Reprinted from reference [52] with permission. (Online version in colour.)

DEHS are elastography techniques that use external or internal harmonic stimuli to generate dynamic responses in biological soft tissues. Here, we focus on cases in which external vibrators or ARFs generated by focused ultrasound beams are used as stimuli. The ARF (figure 3) produced by the momentum transfer from acoustic waves to the medium is determined by

2.1 $$f=\frac{2\alpha I}{c_{L}},$$

where *α* (dB/m) and *c*_L_ (m s^−1^) denote the acoustic absorption and sound speed in the target biological soft tissues, respectively, and *I* (W m^−2^) is the temporal average intensity of the acoustic beam [18,52,53]. *f* (N m^−3^) is a type of body force, and its direction is along the acoustic wave propagation direction. Sarvazyan *et al*. [18] argued that the ARF provides physicians with a ‘virtual finger’ that helps them to touch the internal regions of human bodies.

In DEHS, the steady-state responses of the target soft tissues are typically used to infer their mechanical properties. DEHS may include sonoelastography [16,19,54–58], shear wave-induced resonance elastography (SWIRE) [59–61], vibro-acoustography (VA) [62–67], harmonic motion imaging (HMI) [68–73] and shear wave dispersion ultrasound vibrometry (SDUV) methods [74–79]. The first two methods use external vibrators, while the last three use the ARFs as harmonic stimuli. A brief overview of these methods is given below.

#### (i) Sonoelastography

**Figure 4.**
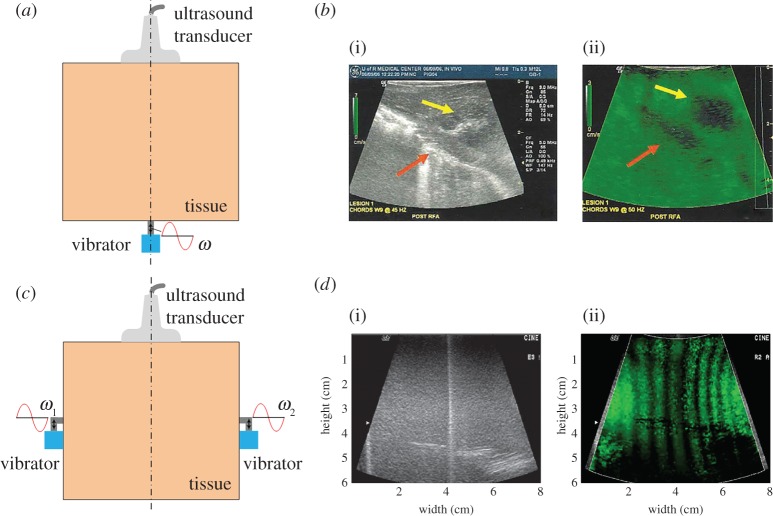
(*a*) Schematic of sonoelastography. (*b*) The B-mode image (i) and vibration amplitude map (ii) obtained via sonoelastography of a porcine liver with a thermal lesion [5]. (*c*) Schematic of crawling wave imaging. (*d*) The B-mode image (i) and a frame showing the crawling waves (ii) in a phantom consisting of a hard layer (left) and soft layer (right) [81]. Reprinted from references [5] and [81] with permission. (Online version in colour.)

The sonoelastography imaging method uses an external harmonic vibrator to generate harmonic vibrations within target soft tissues [5,16,56,58,80]. A schematic of the sonoelastography method is shown in figure 4*a*. The steady-state responses (e.g. the map of the vibration amplitude [55,56] and phase [16]) of the tissues are then measured using the Doppler spectrum of the reflected signals [82,83]. Accordingly, different inverse approaches are established to obtain the elastogram of the tissues. Based on the distribution of the vibration amplitude, local lesions, which may be harder or softer than surrounding tissues, can be distinguished [55,80]. According to the map of the phase, the phase velocity *c* of the shear wave can be measured using a phase gradient algorithm, i.e.

2.2 $$c=\frac{\omega }{\Delta \varphi /\Delta r},$$

where the phase gradient is calculated by the phase shift Δ*φ* over a distance Δ*r* and *ω* denotes the angular frequency. Therefore, the elastic properties of the tissues may be determined [16,58]. The two approaches mentioned above are named vibration amplitude sonoelastography and vibration-phase gradient sonoelastography, respectively [5]. In general, vibration amplitude sonoelastography is a qualitative method that mainly provides information about the positions of local lesions, whereas vibration-phase gradient sonoelastography may be used to quantitatively measure the elastic and viscous properties of soft tissues. Figure 4*b* shows the B-mode image and vibration amplitude map of a porcine liver with a thermal lesion. This figure shows that the lesion can be clearly distinguished from the vibration amplitude map obtained via sonoelastography. Indeed, using vibration-phase gradient sonoelastography, the *in vivo* elastic properties of skeletal muscles [58] and livers [54] of healthy volunteers have been determined.

Later on, an improved method based on the sonoelastography, named crawling wave imaging, was developed by Wu *et al*. [57,81]. The key concept is presented in figure 4*c*. Two vibrators with frequencies of *ω*_1_ and *ω*_2_, respectively, are used to generate shear waves: *ω*_1_ = *ω* + *δω*/2 and *ω*_2_ = *ω* − *δω*/2, where *δω*/*ω* ≈ 0.01 and *ω* is typically hundreds of hertz. The shear waves generated by the two vibrators form an interference pattern that propagates with velocity (*δω*/2*ω*)*c*, where *c* is the phase velocity of the shear wave at frequency *ω* in the soft tissue. Because *δω*/*ω* ≈ 0.01, the velocity of the travelling interference pattern, which is named the ‘crawling wave’, is much smaller than *c* [57,81]. The slow crawling wave can be visualized and tracked by a conventional ultrasonic scanner modified for sonoelastography [84]. The crawling waves in a phantom consisting of a hard layer (left part in (i) and (ii)) and soft layer (right part in (i) and (ii)) are shown in figure 4*d*. Clearly, the wavelength of the crawling wave in the harder region is larger. Initial *ex vivo* experiments on livers and prostates and *in vivo* experiments on skeletal muscles indicate that crawling wave imaging is a promising method for quantitatively characterizing the mechanical properties of biological soft tissues [85–88].

#### (ii) Shear wave-induced resonance elastography

**Figure 5.**
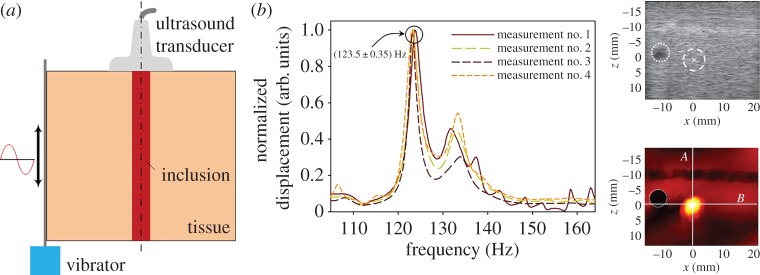
(*a*) Schematic of the SWIRE. (*b*) Typical frequency-domain displacement curves at a point within the soft inclusion, from which the resonance frequency can be determined from the peaks in the curves. The stationary shear wave displacement field in the ROI at the resonance frequency is also presented [61]. Reprinted from reference [61] with permission. (Online version in colour.)

A recently developed method named SWIRE adopts an external vibrator to generate shear waves with frequencies in the range of 45–205 Hz within soft materials, as shown in figure 5*a*. The propagation of the shear waves is monitored by an ultrafast ultrasound scanner. Then, Fourier transformation is conducted for the time-domain displacement at each point in the region- of-interest (ROI) to obtain the frequency-domain displacement. The resonance frequencies of the soft inclusion, which correspond to the low-order eigenmodes of a soft inclusion--hard matrix system, can be identified from the peak values of the frequency-domain displacement curve[59–61]. Additionally, a finite-element (FE) model is used to simulate the experiments and calculate the theoretical resonance frequencies of soft inclusions with different elastic parameters. When the theoretical results match the experimental ones, the corresponding elastic parameters are supposed to be those of the practical soft inclusion. Typical frequency-domain displacement curves for a point within the soft inclusion are shown in figure 5*b*, and from these curves, the resonance frequency may be determined. The stationary shear wave displacement field in the ROI at the resonance frequency is also presented. Clearly, the resonance response of the soft inclusion distinguishes itself from the surrounding harder tissues [59,61]. It should be pointed out that the limitation of SWIRE lies in that it can be only used for the characterization of soft inclusions.

#### (iii) Vibro-acoustography

**Figure 6.**
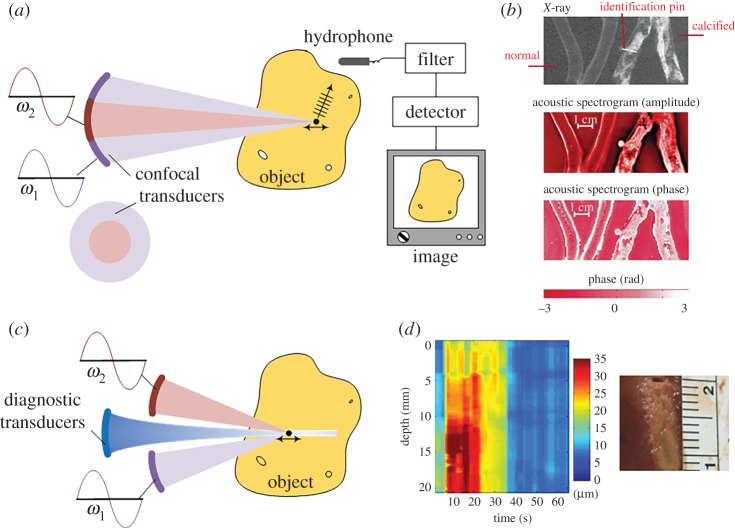
(*a*) Schematic of VA. (*b*) The amplitude and phase acoustic spectrograms of normal and calcified excised human iliac arteries obtained using the VA method [62]. (*c*) Schematic of HMI. (*d*) The HMI displacement variation and corresponding pathology images of liver tissue for 30 s of sonication [71]. Reprinted from references [62] and [71] with permission. (Online version in colour.)

In the VA technique [62–64,89], confocal transducers with centre frequencies *ω*_1_ and *ω*_2_, as shown in figure 6*a*, are applied to a target soft material. *ω*_1_ and *ω*_2_ are on the order of megahertz, whereas *δω* = *ω*_2_ − *ω*_1_ is on the order of kilohertz. Thus, the low-frequency (kHz) harmonic ARF can be imposed onto a focused point within the target soft tissue [62]. The resulting vibration, which is determined by the local elastic properties of the tissue at the focused point, will induce an acoustic emission field. Both the magnitude and phase of the acoustic emission can be detected by a hydrophone. When the two focused ultrasound beams sweep through the whole object, images based on either the magnitude or phase of the acoustic emission can be obtained as shown in figure 6*a*. These images are the so-called magnitude and phase acoustic spectrograms; their resolutions are determined by the ultrasound resolution and the point-spread function of the system, and are roughly hundreds of micrometres [62,63]. Figure 6*b* shows the amplitude and phase images of normal and calcified excised human iliac arteries obtained with *δω* = 6 kHz. Subsequent studies using this technique have demonstrated its promising use in imaging of breast [89,90] and prostate tissues [65,67]. In principle, VA is an interesting imaging technique and has a resolution similar to that of X-ray images when used to measure calcified arteries (figure 6*b*). However, interpreting the acoustic spectrogram to quantitatively determine the mechanical properties of soft tissues is challenging, as recently addressed by Brigham *et al*. [66].

#### (iv) Harmonic motion imaging

To identify the local mechanical properties of soft tissues, Konofagou *et al*. [68–70] modified the experimental set-up of the VA method and proposed the HMI method. In the HMI method, an additional ultrasound beam, as shown in figure 6*c*, is used to monitor the harmonic motion induced by confocal transducers. The amplitude of the harmonic motion of soft tissues is determined by both the local elastic properties at the focus and the magnitude of the ARF. Equation (2.1) shows that the ARF relies on the intensity of the acoustic beam and the acoustical properties of the medium at the focal region; both of them may vary from site to site. Therefore, the magnitude of the ARF is difficult to control, and quantitatively measuring the local elastic modulus is challenging. However, the highly localized harmonic ARF provides a useful way to probe the relative variation of the mechanical properties within biological tissues [69]. Accordingly, the HMI method has been successfully used to monitor HIFU treatment [71,73,91]. Figure 6*d* shows the HMI displacement variation and corresponding pathology images of liver tissue for 30 s of sonication.

In a recent study, Vappou *et al*. [72] developed a two-step inverse approach to quantitatively probe the viscoelastic properties of a soft solid based on HMI. Briefly, the phase velocity of the propagating shear wave induced by the harmonic motion at the focus is measured, and then, the phase shift between the stress (i.e. the harmonic ARF) and the strain (i.e. the monitored harmonic motion) is measured to determine the loss tangent [92]. Hence, the quantitative viscoelastic properties of a soft tissue may be determined. Experiments on phantoms have validated the effectiveness of the inverse approach.

#### (v) Shear wave dispersion ultrasound vibrometry

**Figure 7.**
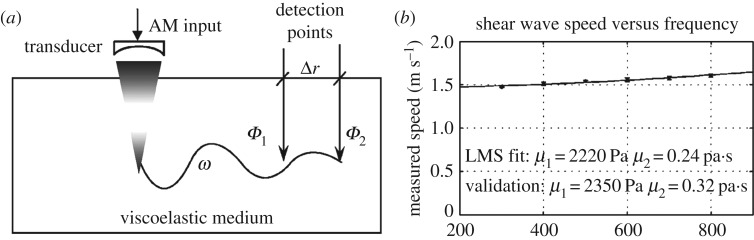
(*a*) Schematic SDUV [74]. (*b*) Dispersion curve of a phantom. The points were obtained from experiments, and the solid line is the least mean square fitting result. The viscoelastic parameters of the phantom determined using the SDUV method agree well with those obtained from an independent measurement [74]. Reprinted from reference [74] with permission.

The SDUV technique, which was developed by Chen *et al*. [74,75], uses the harmonic ARF generated by an amplitude-modulated ultrasound beam to induce low-frequency shear waves (typically ranging from 300 to 900 Hz) within target soft tissues (figure 7*a*). The shear waves are detected at different locations along the propagation direction, and then the phase velocities can be determined via the phase gradient method. For a viscoelastic solid, the phase velocities depend on the frequencies of the shear waves, and the relationship between the phase velocities and the frequencies is the so-called dispersion relation. By controlling the modulation frequency, the frequency of the generated shear wave can approximately vary from 300 to 900 Hz and the dispersion curve can be obtained.

Assuming that the viscoelastic properties of a solid can be described by a Voigt model [92], the theoretical dispersion relation can be derived. Then, by fitting the experimental dispersion curve with the theoretical solution, the elasticity and viscosity parameters can be obtained. A typical experimental dispersion curve for a viscoelastic phantom is shown in figure 7*b*. The elasticity and viscosity parameters of the phantom identified using the SDUV method agree well with those obtained via an independent measurement [74]. Experiments on *in vitro* porcine muscles and human prostates have been conducted to validate the effectiveness of this method [76,93].

A common feature of the aforementioned methods is their reliance on the use of harmonic stimuli to deform soft tissue. By determining the phase velocities of the shear waves, the elastic or viscous parameters of soft tissues may be quantitatively determined. However, when the wavelength of the shear wave is comparable to the dimension of the soft tissue, the phase velocities may depend not only on the physical properties of the soft tissues, but also on the geometrical parameters of the system. In this case, caution should be taken when inferring the tissue parameters based on the shear wave velocities. The SWIRE method uses the resonance frequency information of soft tissues to determine their mechanical properties (e.g. the elastic properties of an inclusion). However, this method relies on the use of a numerical method (e.g. the FE method (FEM)) to simulate the experiments and further evaluate the elastic parameters of an inclusion. This reliance complicates the use of this method in clinics.

### (c) Dynamic elastography with transient stimuli

In this section, we discuss the dynamic elastography methods that use transient stimuli to deform a soft tissue. In these methods, the responses of soft tissues (e.g. the shear wave velocities) are measured to extract the local elastic properties [17,18,20,94–98]. Note that in this method, the frame rate of the scanner is usually high enough to acquire the propagation process of the shear waves. A key merit of DETS is that it is not so sensitive to the BCs. The reflected waves are separated from the incident wave in the time domain and can be filtered out [17]. Therefore, DETS enables the quantitative determination of elastic properties. Similar to DEHS, various external and internal stimuli may be used to generate transient shear waves within soft tissues. In recent years, DETS, in which the ARF is used to generate a transient shear wave, has attracted considerable attention [4,7,18,20,95,96,99,100]. This method, which generates remotely transient shear waves within biological tissues, permits us to probe the mechanical properties of biological tissues and is relatively suitable for clinical use. Here, we focus on the following methods.

#### (i) Transient elastography

**Figure 8.**
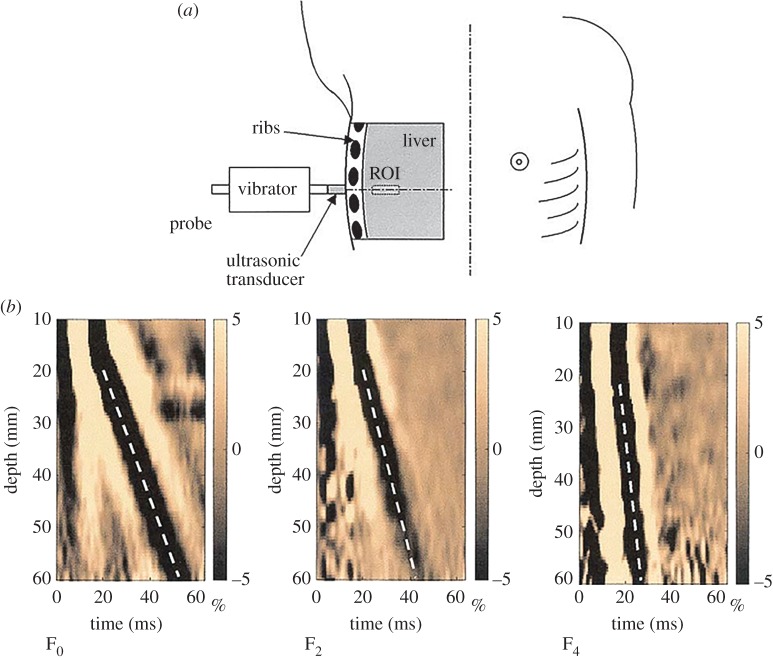
(*a*) Schematic of TE [17]. (*b*) Propagation of the transient elastic waves in livers with different degrees of fibrosis. The results from left to right are for F_0_, F_2_ and F_4_. The velocities of the transient waves can be deduced from the slope of the white dotted lines: a steeper line indicates a faster wave [17]. Reprinted from reference [17] with permission. (Online version in colour.)

Transient elastography (TE), which was proposed by Sandrin and colleagues [17,24,101,102], uses an external vibrator to introduce a low-frequency transient wave in biological tissues and tracks the propagation of this transient wave along the axis of the vibrator (figure 8*a*), with a frame rate of approximately 4000 Hz. The velocity of the transient wave is measured, and the elastic modulus of the soft tissue can be quantitatively determined by assuming that the tested material is elastic and its dimension is larger than the wavelength. The TE technique forms the basis of Fibroscan® (Fibroscan, Echosens™, France), which is an effective approach for staging liver fibrosis [103,104]. Figure 8*b* shows the *in vivo* experimental results obtained from three livers with different degrees of fibrosis (F_0_ to F_4_ denote the degree of fibrosis). Clearly, for a liver with a higher degree of fibrosis (e.g. F_4_), the velocity of the transient wave in the ROI is greater.

#### (ii) Shear wave elasticity imaging and ARF impulse

**Figure 9.**
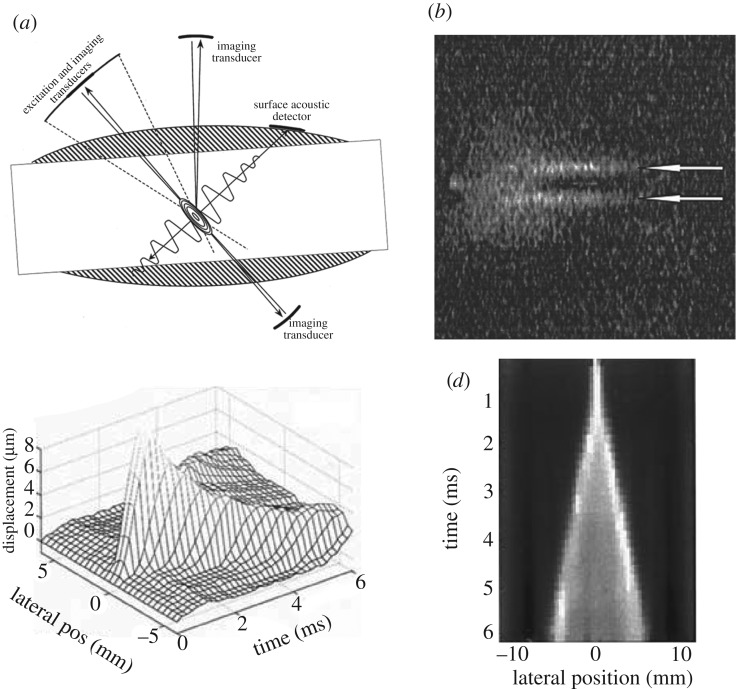
(*a*) Schematic of the SWEI method [18]. (*b*) The shear waves recorded by the MRI [18]. (*c*) The shear waves recorded via the AFRI-based SWEI method, showing the displacements along the lateral direction at different times. Clearly, the displacements are attenuated along the propagation direction [94]. (*d*) Normalized displacement map obtained from (*c*); based on the slope of the bright line, the shear wave velocities can be evaluated [94]. Reprinted from references [18] and [94] with permission.

Sarvazyan *et al*. [18] proposed the shear wave elasticity imaging (SWEI) method, which uses the ARF to generate shear waves within soft tissues (figure 9*a*). The magnitude of the ARF is fairly small, and, thus, the displacement induced by the acoustic force within the soft biological tissue is usually on the order of micrometre. However, Sarvazyan *et al*. [18] demonstrated that using an additional system (e.g. MRI or optical imaging) facilitates recording the shear waves, as shown in figure 9*b*. Subsequently, Nightingale *et al*. [94] and Palmeri *et al*. [98] used ultrasound imaging to track the propagation of shear waves in *in vivo* and *ex vivo* experiments. The experimental set-up used by Nightingale *et al*. [94] is based on their previously developed ARF impulse (ARFI) technique, which has been implemented commercially by Siemens Medical Solutions on their ACUSON S2000^TM^ [21,99]. The ARFI technique imposes the impulse radiation force onto local regions of the tissues, and information, such as the displacements immediately after the excitation, the peak displacement, the time to reach the peak displacement and the recovery time of the deformed region after the force is removed, can be used to determine the local elastic properties of soft tissues based on dedicated inverse approaches [21,105]. Typically, the map of the peak displacement, which is assumed to be inversely proportional to the elastic modulus of the local tissue, is provided to indicate the stiffness distribution within the target soft tissue. Here, the method in which ultrasound imaging is used to track the shear waves induced by ARFI is named the AFRI-based SWEI method [35,94,106]. Figure 9*c* and *d* shows the displacement induced by the AFRI and the normalized displacement used to evaluate the velocity of the shear wave, respectively.

AFRI-based SWEI method [35,94,106] is a promising strategy for quantitatively probing the local elastic properties of biological soft tissues. Both generating the ARF and monitoring the propagation of the shear waves can be realized using a single ultrasound probe, unlike in experimental systems that require an extra vibrator to generate shear waves. Subsequent studies of ARFI-based SWEI have demonstrated its usefulness for *in vivo* characterization of the mechanical properties of various soft biological tissues [35,98,106].

#### (iii) Supersonic shear imaging

**Figure 10.**
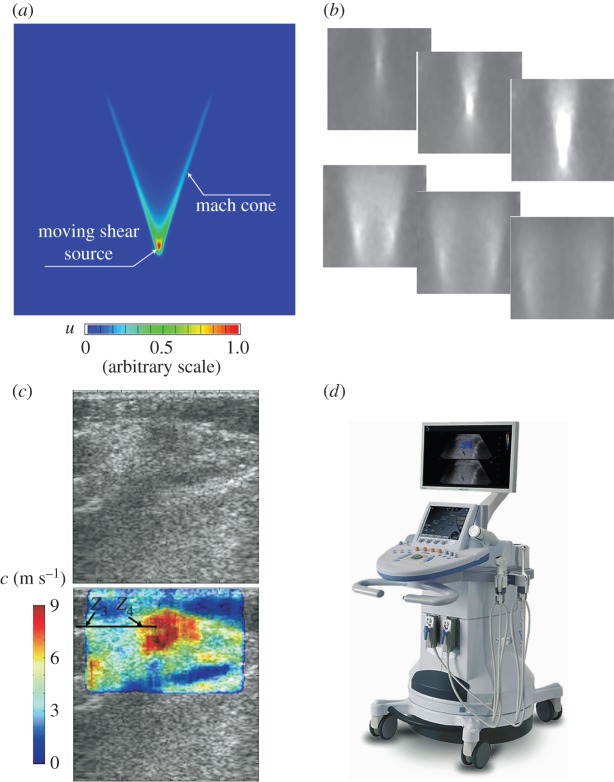
(*a*) The displacement field given by FEA, indicating the formation of the shear-wave Mach cone. In this case, the moving velocity of the vibration source is three times the velocity of the shear wave in the target material. (*b*) The propagation process of the interfered wavefronts within 14 ms after applying the ARF recorded during an experiment on a phantom [20]. (*c*) The *in vivo* elastogram obtained via SSI for breast tissues [95]. (*d*) A photograph of the Aixplorer® ultrasound instrument (Supersonic Imagine, Aix-en-Provence, France). Reprinted from references [20] and [95] with permission. (Online version in colour.)

The supersonic shear imaging (SSI) technique uses ultrasound beams focused at different depths within biological tissues to create a moving ARF [20,95]. The ARF moves at a high speed in the soft material; thus, the resulting displacement field is confined within a Mach cone, as shown in figure 10*a*. In this case, two quasi-plane shear wavefronts interfere along the Mach cone and propagate in opposite directions. This phenomenon is known as the elastic Cherenkov effect (ECE) [20,34,107]. In the SSI technique, the propagation of the interfered front is monitored using an ultrafast imaging technique. The fast acquisition reduces the risk of artefacts resulting from the movements of patients or investigators. Typical experimental results for a homogeneous phantom are shown in figure 10*b*, which illustrates the propagating process of the interfered wavefronts within 14 ms after imposing the moving ARF. Furthermore, the shear wave velocity is measured using the time-of-flight algorithm [95,108], and the elastic moduli of the target soft tissues can be determined. Figure 10*c* shows an *in vivo* elastogram obtained in breast tissues. The central region of the ROI (the red region), in which Young's modulus is higher than in the surrounding tissues, is suspected to be a lesion. The SSI technique has now been commercialized (figure 10*d*) and used in clinics. Applications of this technique to detect breast lesions [95,109] and thyroid nodules [110] and stage liver fibrosis [111] have been explored.

Moreover, determination of the nonlinear elastic properties of soft tissues using the SSI technique has attracted considerable attention in recent years [8,26–28,33,34]. In these studies, the tested soft materials are pre-deformed, and then the shear wave velocities in the deformed materials are measured. According to the relationship between the velocities of shear waves and the pre-deformation and material parameters, the nonlinear elastic properties can be determined. The nonlinear elastic properties for human breasts, heel fat pads and skeletal muscles have been measured *in vivo* [8,33] in this way. To evaluate nonlinear elastic properties using these methods, the pre-deformation must be determined with a reasonable accuracy. Li and colleagues [8,27,33] evaluated the pre-deformation based on B-mode images. In a recent study, Bernal *et al*. [28] developed an experimental system in which the pre-deformation is evaluated using the static elastography method, and the shear wave velocities are measured using the SSI technique. Thus, the two-dimensional images of the nonlinear elastic properties can be obtained. They argued that the map of the nonlinear elastic properties might produce better contrast between lesions and normal soft tissues [28].

#### (iv) Comb-push ultrasound shear elastography

**Figure 11.**
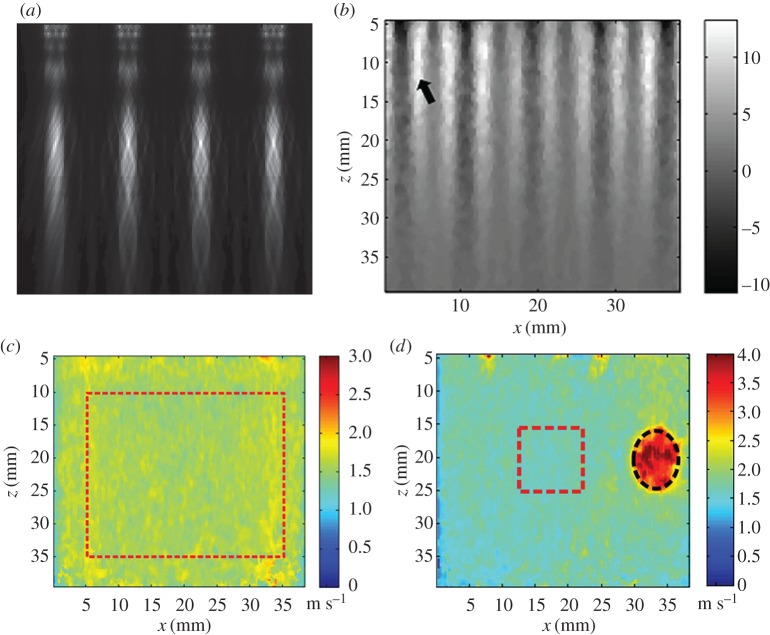
(*a*) ARFs generated by multi unfocused ultrasound beams. (*b*) Shear waves generated by CUSE in a homogeneous phantom. Panels (*c*) and (*d*) give the shear wave velocity maps in the whole FOV for a homogeneous phantom and a phantom with a hard inclusion, respectively [96]. Reprinted from reference [96] with permission. (Online version in colour.)

Song *et al*. developed the comb-push ultrasound shear elastography (CUSE) method [96,112–114]. In this method, several unfocused (or focused) ultrasound beams as shown in figure 11*a*, are used as multi-stimuli to generate shear waves in the whole field-of-view (FOV). Additionally, the elastic properties in the whole FOV can be identified with one acquisition. Figure 11*b* shows the shear waves generated by the CUSE in a homogeneous phantom. Both homogeneous and inclusion phantom experiments have been performed to validate the effectiveness of this method, as shown in figure 11*c* and *d*. *In vivo* experiments to detect breast masses and evaluate thyroid nodules have also been conducted, and the results show that this technique is promising [115,116]. In the subsequent study, Song *et al*. further developed the time-aligned sequential tracking (TAST) method, which enables the CUSE being realized on traditional ultrasound scanner [113].

#### (v) Guided wave elastography

When using the dynamic elastography methods mentioned above, body wave theories are typically used to relate the shear wave velocities to the material parameters of soft issues. However, when dynamic elastography methods are used to characterize thin-walled soft tissues, the waves are guided, and, therefore, the guided wave theory should be used to interpret the experimental data [100,117–122]. Guided wave elastography (GWE) methods have attracted increasing interest in recent years, although they have not been commercialized yet. For thin-walled soft tissues, the wall thickness may be smaller than or comparable to the wavelength of the elastic waves generated in soft tissues; in this case, the waves are guided within the wall and are strongly dispersive [123,124]. The dispersion relation is crucial for inferring the material parameters from the measured experimental responses.

The general steps involved in GWE using the ARF as stimuli may be summarized as follows.

(1) The focused ARF is used to generate the broad-band guided waves in the walls of soft tissues.
(2) The propagation of the guided shear waves is tracked along the propagation direction.
(3) Two-dimensional Fourier transformation can be applied to analyse the spatio-temporal imaging of the guided waves and extract the dispersion relation [125].
(4) A guided wave model (e.g. the Lamb wave model [123]) can be used to fit the experimental dispersion curve and identify the elastic properties of the thin-walled soft tissues.

**Figure 12.**
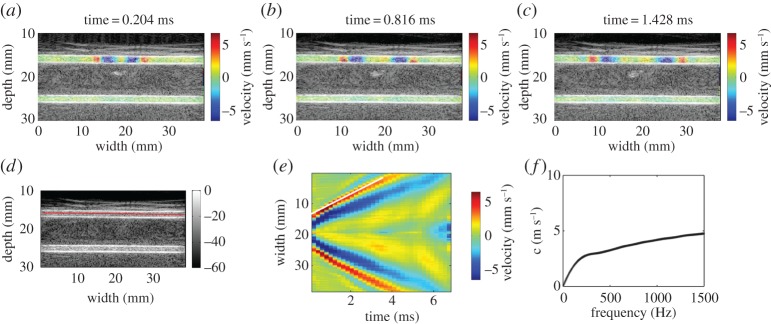
(*a*–*c*) The axial components of the partical velocities in a vessel-mimicking phantom overlaid on the B-mode image at approximately 0.2, 0.8 and 1.4 ms after the action of the ARF. The inner radius and wall thickness of the vessel-mimicking phantom are 2 mm and 1 mm, respectively. (*d*) The solid line along which the spatio-temporal imaging of the shear wave propagation occurred is shown in (*e*). (*f*) The dispersion curve derived from spatio-temporal imaging via a two-dimensional Fourier transformation [119]. (Online version in colour.)

Typical experimental results of a vessel-mimicking phantom are shown in figure 12, in which the wave propagates along the axial direction of the vessel. From the dispersion curve given in figure 12*f*, the elastic properties of the vessel-mimicking phantom can be determined by using the guided wave model. *Ex vivo* and *in vivo* experiments performed on arteries [117,118], bladders [126], tendons [120] and heart wall [121] demonstrate that the GWE method is a promising tool for measuring the elastic properties of thin-walled biological tissues.

The key issue affecting the use of the GWE method is the development of robust inverse approaches based on the dispersion relations given by appropriate guided wave models. The dispersion relation is generally sensitive to the BCs, geometrical parameters and pre-stresses in the soft tissues [100,117–119,127,128], which should be addressed during the development of a robust inverse method.

## 3. Mechanics underpinning ultrasound-based elastography

Continuum mechanics plays an essential role in the development, evaluation and improvement of both static elastography and dynamic elastography methods. From the viewpoint of direct analysis, continuum mechanics enables the prediction of the responses of a biological soft tissue under either a static load or a dynamic stimulus. For instance, in static elastography, continuum mechanics predicts that the softer part will undergo larger deformation than the stiffer part. Therefore, measuring the deformation of soft tissues using a medical imaging method enables differentiating the parts with different elastic moduli. In dynamic elastography, understanding the correlation between the dynamic responses of soft tissues and their mechanical properties under either harmonic or transient stimuli within the framework of continuum mechanics forms the basis of developing data analysis methods to infer the material parameters of soft tissues. Moreover, inferring the mechanical properties of a biological soft tissue based on the responses of the soft tissue to an external or internal stimulus represents an inverse problem in elasticity. Unlike direct problems, many important inverse problems in engineering and science are ill-posed [129]. An inverse problem is ill-posed if one of the following properties is not respected. (1) A solution to the problem exists (existence). (2) There is, at most, one solution to the problem (*uniqueness*). (3) The solution depends continuously on the data (*stability*). To identify the extent to which the material parameters of a soft tissue can be effectively inferred using an elastography method, the properties of the inverse problem (i.e. the existence, uniqueness and stability of the solution) should be addressed by invoking the mathematical theory of inverse problems. In this section, we first summarize the governing equations involved in the use of the aforementioned elastography methods to characterize the mechanical properties of soft tissues. Then, the specific mechanics models for different types of elastography methods are discussed. Particular attention is paid to the theoretical solutions that describe the correlations between the experimental responses and material parameters, and their limitations are emphasized.

### (a) Governing equations

#### (i) Equilibrium equations

The equilibrium equation describing the conservation of momentum [130,131] is given by

3.1 $$\frac{\text{d}}{\text{d}t}\int_{V}\rho \frac{\text{d}\text{u}}{\text{d}t}\text{d}V=\oint_{S}\text{T}\text{d}S+\int_{V}\rho \text{b}\text{d}V.$$

In the above equation, **u** denotes the displacement of the elastic solid, *t* denotes time, *ρ* denotes the mass density and **b** is the body force. **T** is the surface traction force and is related to the Cauchy stress σ by

3.2 T=σ⋅n,

where **n** denotes the outer unit normal. Using the divergence theorem, we can obtain the differential form of equation (3.1)

3.3 $$\rho \frac{{\text{d}}^{2}\text{u}}{\text{d}t^{2}}=\nabla \cdot \sigma +\rho \text{b}.$$

The above equation represents the conservation of linear momentum, and the conservation of the angular momentum further leads to the symmetry of σ; i.e. $\sigma =\sigma^{T}$ [132].

#### (ii) Kinematic equations

Here, we use $B_{r}$ and B to denote the reference (undeformed) and current (deformed) configurations, respectively; the points related to $B_{r}$ and B are labelled using vectors $\text{X}=X_{\alpha }{\text{E}}_{\alpha }$ and $\text{x}=x_{i}{\text{e}}_{i}$ (α,i∈{1,2,3}, respectively, where the Roman and Greek indices refer to the configurations B and $B_{r}$, respectively. The displacement **u** is u=x−X, and the deformation gradient tensor **F** is defined as

3.4 $$\text{F}=\frac{\partial \text{x}}{\partial \text{X}}=\frac{\partial \text{u}}{\partial \text{X}}+\text{I}.$$

The determinant of **F**, which is denoted as *J*, gives the local volume ratio between the deformed and undeformed configurations. The soft biological tissues considered in this study are typically assumed to be incompressible in the literature (i.e. *J* = 1). Furthermore, the Green strain tensor can be defined as

3.5 $$\text{E}=\frac{1}{2}(\text{C}-\text{I}),$$

where **C** is the right Cauchy–Green deformation tensor

$$\text{C}={\text{F}}^{T}\cdot \text{F}.$$

Inserting equations (3.4) and (3.6) into equation (3.5) gives

3.7 $$\text{E}=\frac{1}{2}[\nabla_{r}\text{u}+{\left(\nabla_{r}\text{u}\right)}^{T}+(\nabla_{r}\text{u})\cdot {\left(\nabla_{r}\text{u}\right)}^{T}],$$

where ∇_r_() stands for the gradient operator in $B_{r}$.

When the deformation is infinitesimal, the difference between $B_{r}$ and B can be ignored, and the Green strain tensor can reduces to the small-strain tensor

3.8 $$\varepsilon =\frac{1}{2}(\nabla \text{u}+\text{u}\nabla ).$$

The amplitudes of the shear waves used in the elastography techniques are much smaller than the feature size of the tissues [4]. Moreover, the static strain used in the static elastography technique is small, and typically, the axial strain is of the order of 2% [6,15]. However, in many cases, the target soft tissues may be pre-loaded (say by the ultrasound probe) before the imaging process, and finite deformations may occur (e.g. the strain may reach 10–20%). Such a pre-load is necessary and can help to increase the contrast, reduce the decorrelation noise [3,46], and measure the nonlinear elastic properties of soft biological tissues [8,26–28]. In all of these cases, the problem can be summarized as ‘small on large’, that is, the small deformation caused by the shear wave or the static compression is superimposed on the finite deformation caused by the pre-load. To address the effect of the pre-deformation, the finite elasticity and incremental theory should be applied [131,133,134].

#### (iii) Constitutive laws

For a linear elastic and isotropic solid, the linear constitutive law is given by

3.9 σ=Cε,

where C is the fourth-order elasticity tensor. Inserting equation (3.8) into equation (3.9), we can obtain the linear constitutive law in component form as

3.10 $$\sigma_{ij}=C_{ijkl}u_{k,l},$$

where i,j,k,l∈{1,2,3}. In the most general case, C has 21 independent components [130]. For an isotropic elastic solid, the number of independent components of C reduces to 2, and in this case, using the kinematic relations given by equation (3.8), equation (3.9) reduces to

3.11 σ=μ(∇u+u∇)+λ(∇⋅u)I,

where *λ* and *μ* are the Lamé constants and are related to the elastic modulus *E* and Poisson ratio *ν* through *μ* = *E*/[2(1 + *ν*)] and *λ* = *νE*/[(1 + *ν*)(1−2*ν*)]. For most soft biological tissues, *ν* is close to 0.5, and thus λ≫μ and *μ* ≈ *E*/3.

For the elastography of anisotropic soft tissues, here, we mainly concentrate on the transversely isotropic (TI) model, which is typically used to model anisotropic biological tissues, such as skeletal muscles and tendons [135,136]. In the TI model, C has five independent components, and this number further reduces to three with the constraint of incompressibility [137]. Usually, the three elastic parameters, namely *μ*_T_, *μ*_L_ and *E*_L_, can be used as three independent parameters to fully describe the mechanical properties of incompressible TI materials [34]. In general, *μ*_T_ and *μ*_L_ denote the transverse and longitudinal shear moduli, respectively, and *E*_L_ is the longitudinal elastic modulus. Details about the relationships between *μ*_T_, *μ*_L_ and *E*_L_ and the components of C can be found in [34,35].

To describe the nonlinear elastic deformation of soft tissues, constitutive laws are usually defined using the strain energy function *W*, which is a scalar function of the strain invariants [131,138]. Then, the nominal stress **S**, which is defined as $\text{S}=J{\text{F}}^{-1}\cdot \sigma$, can be determined by

3.12 $$\text{S}=\frac{\partial W}{\partial \text{F}}-p{\text{F}}^{-1},$$

where *p* is a Lagrange multiplier used to ensure the incompressibility constraint [131,133].

For an isotropic solid, the first two principal invariants of **C**, which are denoted as *I*_1_ and *I*_2_, are

3.13 $$I_{1}=\text{tr( }\text{C}\text{) },\;I_{2}=\frac{1}{2}[I_{1}^{2}-\text{tr(}{\text{C}}^{2}\text{) }].$$

Because of the constraint of incompressibility, the third invariant *I*_3_ = 1, and *W* = *W*(*I*_1_,*I*_2_). Here, we list several hyperelastic models that have been widely used in the literature.

The neo-Hookean model is a simple and broadly used hyperelastic model, and its strain energy density function is given by

3.14 $$W=\frac{\mu_{0}}{2}(I_{1}-3),$$

where *μ*_0_ is the initial shear modulus. In acoustoelasticity theory, the following fourth-order strain energy function has been used by many authors [26,139]

3.15 $$W=\mu_{0}\;\text{tr(}{\text{E}}^{2}\text{) }+\frac{A}{3}\;\text{tr(}{\text{E}}^{3}\text{) }+D{\left(\text{tr(}{\text{E}}^{2}\text{) }\right)}^{2},$$

where *A* and *D* are the third- and fourth-order Landau constants, respectively, describing the nonlinear elastic deformation behaviours of soft materials.

To describe the nonlinear elastic deformation of a soft tissue, Demiray [140] and Fung et al. [141] proposed a strain energy density function in the following form:

3.16 $$W=\frac{\mu_{0}}{2b}({\text{e}}^{b(I_{1}-3)}-1),$$

where the parameter *b* > 0 is linked to the hardening effect [8,27].

For an anisotropic material, the material characteristic directions in the reference configuration can be denoted by the unit vectors **A***_α_* (*α* = 1, 2, … , *N*)). These characteristic directions may be caused by fibre-reinforced effects, such as spatially oriented collagen fibres [138,142]. In this case, some other invariants must be introduced to define *W*. Following the definitions used in the literature and commercial FE software [143], the following invariants, denoted as *I*_4(*αα*)_ and *I*_5(*αα*)_ (no sum on *α*), are defined:

3.17 $$I_{4(\alpha \alpha )}={\text{A}}_{\alpha }\cdot \text{C}\cdot {\text{A}}_{\alpha }\;\text{and}\;I_{5(\alpha \alpha )}={\text{A}}_{\alpha }\cdot {\text{C}}^{2}\cdot {\text{A}}_{\alpha }.$$

The Holzapfel–Gasser–Ogden model has been widely used to model the nonlinear elastic deformation behaviour of arterial walls [138,142,143], for which the strain energy density function is

3.18 $$W=C_{10}(I_{1}-3)+\frac{k_{1}}{2k_{2}}\sum_{\alpha =1}^{N}\left\{\exp[k_{2}{\left(\kappa (I_{1}-3)+(1-3\kappa )(I_{4(\alpha \alpha )}-1)\right)}^{2}]-1\right\},$$

where *C*_10_, *k*_1_ and *k*_2_ are material constants, and *κ* is defined as

3.19 $$\kappa =\frac{1}{4}\int_{0}^{\pi }\rho (\Theta )\sin^{3}\Theta \text{d}\Theta ,$$

where *ρ*(*Θ*) is the orientation density function which characterizes the distribution of the fibres.

In particular, for TI hyperelastic materials (i.e. those in which the fibres run along only one direction), *N* = 1. In this case, Murphy [144] noted that *W* should include both *I*_4(11)_ and *I*_5(11)_ to ensure compatibility between the linear elastic and nonlinear elastic models, and suggested that *W* may be written as

3.20 $$W=F(I_{1},I_{4(11)})+\frac{\mu_{T}-\mu_{L}}{2}(2I_{4(11)}-I_{5(11)}-1).$$

Furthermore, the following function, which generalizes the Humphrey–Yin model [145], has also been suggested [144]

3.21 $$W=\frac{\mu_{T}}{2c_{2}}[{\text{e}}^{c_{2}(I_{1}-3)}-1]+\frac{E_{L}+\mu_{T}-4\mu_{L}}{2c_{4}}[{\text{e}}^{c_{4}{\left(I_{{\;}_{4(11)}}^{1/2}-1\right)}^{2}}-1]+\frac{\mu_{T}-\mu_{L}}{2}(2I_{4(11)}-I_{5(11)}-1),$$

where *c*_2_ > 0 and *c*_4_ > 0 are the isotropic and anisotropic strain-hardening parameters, respectively.

#### (iv) Incremental theory

Here, we give a brief overview of the incremental deformation theory, which is involved in finite deformation analysis. The finite deformation from the referenced (undeformed) configuration $B_{r}$ to the current (deformed) configuration B is described by the deformation gradient tensor **F**, and the small incremental deformation from the deformed configuration B to $\dot{B}$ is denoted as $\dot{\text{F}}$, where $\left(\dot{\;}\right)$ denotes the small increment from B to $\dot{B}$. As mentioned above, the deformation from B to $\dot{B}$ is assumed to be infinitesimal, and, thus, the two configurations B and $\dot{B}$ are very close to each other.

The incompressible constraint *J* = det(**F**) = 1 in the incremental form is

3.22 $$\text{tr}({\dot{\text{F}}}_{0})=0,$$

where ${\dot{\text{F}}}_{0}=\dot{\text{F}}\cdot {\text{F}}^{-1}=\partial \dot{\text{u}}/\partial \text{x}$, $\dot{\text{u}}$ denotes the incremental displacement.

Inserting **S** into the equilibrium equation (3.3) and noticing that $\nabla_{r}\cdot (J{\text{F}}^{-1})=0$ [131], we obtain

3.23 $$\rho \frac{\partial^{2}\text{u}}{\partial t^{2}}=\nabla_{r}\cdot \text{S}+\rho \text{b}.$$

The incremental form of equation (3.23) is

3.24 $$\rho \frac{\partial^{2}\dot{\text{u}}}{\partial t^{2}}=\nabla_{r}\cdot \dot{\text{S}},$$

where we assume that **ḃ **= 0. From equation (3.12), we obtain

3.25 $$\dot{\text{S}}=A\cdot \cdot \dot{\text{F}}-\dot{p}{\text{F}}^{-1}+p{\text{F}}^{-1}\cdot \dot{\text{F}}\cdot {\text{F}}^{-1},$$

where $A=\partial^{2}W/\partial \text{F}\partial \text{F}$ or, in component form, $A_{\alpha i\beta j}=\partial^{2}W/\partial F_{i\alpha }\partial F_{j\beta }$. To push $\dot{\text{S}}$ into configuration B, we further define the update incremental stress ${\text{S}}_{0}=J^{-1}\text{F}\dot{\text{S}}$. Then, we have

3.26 $$\nabla_{r}\cdot \dot{\text{S}}=J^{-1}\nabla \cdot {\text{S}}_{0}.$$

Equation (3.25) can be rewritten as

3.27 $${\text{S}}_{0}=A_{0}\cdot \cdot {\dot{\text{F}}}_{0}-\dot{p}\text{I}+p{\dot{\text{F}}}_{0},$$

where

3.28 $$A_{0piqj}=J^{-1}F_{p\alpha }F_{q\beta }A_{\alpha i\beta j}.$$

Inserting equation (3.27) into (3.26) and recalling the incompressible constraint given by tr(**Ḟ**_0_) = 0, the updated form of equation (3.24) can be obtained

3.29 $$\rho \frac{\partial^{2}\dot{\text{u}}}{\partial t^{2}}=\nabla \cdot (A_{0}\cdot \cdot {\dot{\text{F}}}_{0})-\nabla \dot{p}+\nabla p\cdot {\dot{\text{F}}}_{0}.$$

The above equation together with the incremental incompressible constraint given by equation (3.22), is the governing equation for the incremental motion.

### (b) Mechanics of static elastography

In static elastography, the deformation and strains are assumed to be small, and the linear elasticity theory is typically used. The specific mechanical model is briefly introduced here based on the governing equations in §3*a*. Inserting equation (3.11) into equation (3.3) and ignoring the body force and inertia force, we obtain

3.30 $$(\lambda +\mu )\nabla (\nabla \cdot \text{u})+\mu \nabla^{2}\text{u}=0.$$

**Figure 13.**
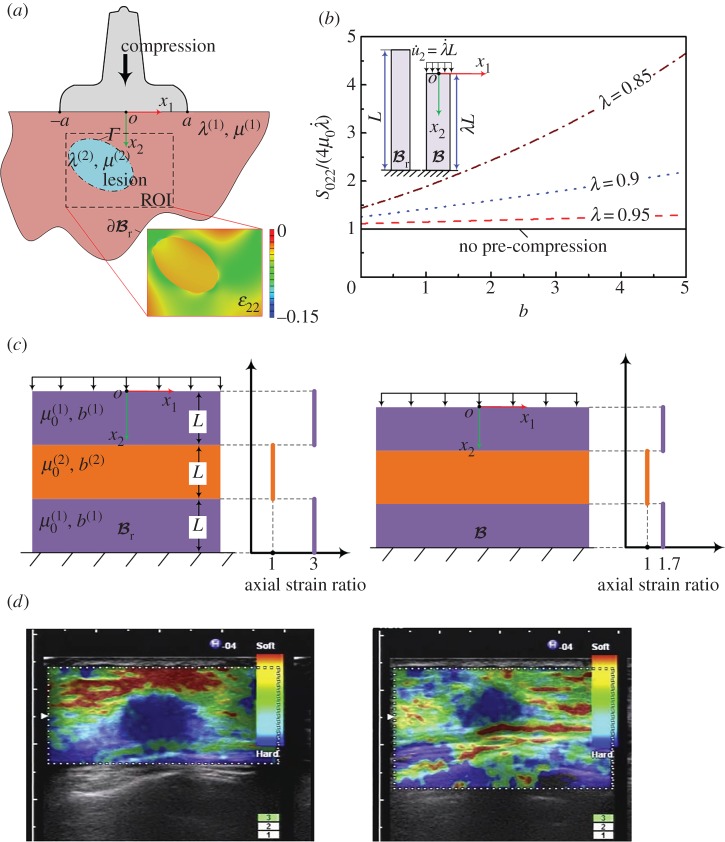
(*a*) Schematic of the mechanical model underlying static elastography and the axial strain in the ROI calculated by FEA (${\dot{u}}_{2}=0.1a$). The inclusion in the ROI is harder than the surrounding tissue. (*b*) Effect of pre-compression on the elastic modulus measured via static elastography. (*c*) Illustration of the decrease in the axial strain image contrast caused by pre-compression. The ratio of the initial shear moduli of the two materials is *μ*^(2)^/*μ*^(1)^ = 3. A 10% pre-compression will cause the strain ratio to decrease from 3 to 1.7. (*d*) The decrease of the strain image contrast due to the excessive compression observed in the experiment of Shiina *et al*. [39]. Reprinted from reference [39] with permission. (Online version in colour.)

As shown in figure 13*a*, here, we take an inclusion buried in surrounding soft tissues as an example. For simplicity, we consider a plane strain problem (i.e. the materials cannot deform in the *x*_3_-direction) [48]. The Lamé constants of the surrounding tissue and the inclusion are *λ*^(1)^, *μ*^(1)^, and *λ*^(2)^, *μ*^(2)^, respectively, where the superscripts ‘(1)’ and ‘(2)’ denote the surrounding tissue and the inclusion, respectively. When the tissue is slightly compressed relative to the undeformed configuration $B_{r}$, the normal displacement at the contact region (−*a* < *x*_1_ < *a*, 2*a* is the width of the ultrasound probe) between the probe and the skin is described as ${\dot{u}}_{2}$, whereas the tangential displacement is not constrained. Therefore, the BCs on *x*_2_ = 0 are

3.31 $$\begin{array}{rl} u_{2}^{\left(1\right)} & ={\dot{u}}_{2},\;\sigma_{12}^{\left(1\right)}=0,\;(-a<x_{1}<a)\\ \;\text{and}\;\sigma_{11}^{\left(1\right)} & =0,\;\sigma_{12}^{\left(1\right)}=0,\;(x_{1}<-a\text{ or }a<x_{1}). \end{array}\}$$

The boundary $\partial B_{r}$ is assumed to be fixed, i.e.

3.32 $$u_{1}^{\left(1\right)}=0,u_{2}^{\left(1\right)}=0,\;\text{on }\partial B_{r}\text{.}$$

At the interface *Γ* between the inclusion and the surrounding tissue, the two materials are assumed to be tied together; therefore, the interfacial conditions (ICs) are

3.33 $$\begin{array}{r} u_{1}^{\left(1\right)}=u_{1}^{\left(2\right)},u_{2}^{\left(1\right)}=u_{2}^{\left(2\right)},\\ \sigma_{12}^{\left(1\right)}=\sigma_{12}^{\left(2\right)},\sigma_{22}^{\left(1\right)}=\sigma_{22}^{\left(2\right)}, \end{array}\},\;\text{on }\Gamma \text{.}$$

With the BCs and the ICs, the direct problem given by equation (3.30) can be solved either theoretically or numerically, such as via FE. In figure 13*a*, the map of *ϵ*_22_ within the ROI is calculated for illustration. The distribution of the axial strain within the biological tissues/organs provides physicians with useful information regarding the homogeneity of the tissues/organs. As shown in figure 13*a*, the strain contrast (i.e. the low compression strain within the elliptical area) obviously indicates the existence of a hard inclusion. The use of elastography to obtain axial strain maps has found wide applications in clinical diagnosis [14,42,45,47].

Although the axial strain can reflect the stiffness distribution in soft tissues, quantitatively measuring the elastic properties of soft tissues using static elastography by solving the inverse problem remains challenging [6,41,48]. This is because, in static elastography, the strain field in the soft tissue depends not only on the physical parameters of the system, but also on the BCs and ICs, which are difficult to accurately determine in most cases. In the literature, methods for obtaining maps of the relative elastic moduli (e.g. the shear moduli ratio *μ*^(2)^/*μ*^(1)^) have been investigated [41,43,44,48,146–148], and readers can refer to the review paper by Doyley for more details [6] regarding the challenges and usefulness of determining *μ*^(2)^/*μ*^(1)^. Assuming that the inclusion is a small cylinder embedded in an infinite matrix and that the composite is compressed, Kallel *et al*. [48] obtained

3.34 $$\frac{1}{P}=\left[\frac{\left(1-2\nu \right)}{Q+(1-2\nu )}+\frac{2}{1+Q(3-4\nu )}\right],$$

where *Q* = *μ*^(2)^/*μ*^(1)^, $P={\bar{\varepsilon }}_{22}^{\left(1\right)}/{\bar{\varepsilon }}_{22}^{\left(2\right)}$, and *ν* is the Poisson ratio of both the inclusion and the matrix. Note that ${\bar{\varepsilon }}_{22}^{\left(1\right)}$ and ${\bar{\varepsilon }}_{22}^{\left(2\right)}$ denote the axial strain far from the inclusion and the axial strain at the centre of the cylinder [48], respectively. Using this simple relation, the moduli ratio *μ*^(2)^/*μ*^(1)^ can be determined from the measured axial strain ratio [147].

Another important issue affecting the practical use of static elastography is the effect of nonlinear elasticity. Indeed, in many cases, the pre-compressions were used to improve the image contrast [3,46]. To illustrate this issue, here, we use static incremental theory to analyse the deformation of a soft tissue. In this case, the incremental motion, which is used to compress the pre-deformed tissue and obtain the axial strain image, is quasi-static; i.e. $\partial^{2}\dot{\text{u}}/\partial t^{2}=0$. We consider a homogeneous tissue modelled using the Demiray–Fung model [140,141] and pre-compressed along the *x*_2_-direction (figure 13*b*). The deformation gradient tensor is homogeneous and denoted as **F **= diag(*λ*^−1^,*λ*,1), where *λ* is the stretch ratio along the *x*_2_-axis (i.e. the axial direction). In this case, the incremental stress can be determined using equation (3.27) and is given by

3.35 $$S_{022}=\mu_{0}\lambda {\text{e}}^{b(\lambda^{2}+\lambda^{-2}-2)}[2b{\left(\lambda -\lambda^{-3}\right)}^{2}+(1+3\lambda^{-4})]\dot{\lambda },$$

where $\dot{\lambda }$ denotes the incremental stretch ratio (figure 13*b*). When *λ* = 1 (i.e. there is no pre-compression), equation (3.35) reduces to $S_{022}=4\mu_{0}\dot{\lambda }$. However, when pre-compression is applied, it stiffens the tissues. For illustration, the variation of the dimensionless stiffness $S_{022}/(4\mu_{0}\dot{\lambda })$ with the hardening parameter *b* in the Demiray–Fung model [140,141] for different amounts of pre-compression is plotted in figure 13*b*. Clearly, the pre-compression results in the overestimation of the tissues stiffness, especially for larger *b*. For different biological tissues, such as brain, liver and breast, *b* varies over a wide range (e.g. from 0.2 to 4.5) [8,27]. In the static elastography of breast tissues, whose hardening parameter *b* is approximately 3.0, the pre-compression strain used in the measurement may be as high as 10% [149]. According to figure 13*b*, in this case, Young's modulus may be overestimated by approximately 60%. To reduce the risk of misdiagnosis [39,150], choosing a proper level of pre-compression is crucial, which requires knowledge of the nonlinear elastic parameters of soft tissues (e.g. the hardening parameter *b* in the Demiray–Fung model).

To further illustrate the effects of pre-compression on the axial strain image contrast, we consider a simple multi-layered model, as shown in figure 13*c*. In this model, a stiff layer is embedded in two soft layers. The initial shear modulus of the stiff layer is denoted as $\mu_{0}^{\left(2\right)}$, and $\mu_{0}^{\left(1\right)}$ is the initial shear modulus of the soft layers. The hardening parameters of the stiff layer and softer layers are denoted as *b*^(2)^ and *b*^(1)^, respectively. For illustration, we take *b*^(1)^ *=* *b*^(2)^ = 3. When an overall pre-compression is applied to the composite, the compression strain in the soft layers is greater than that in the stiff layer because $\mu_{0}^{\left(2\right)}>\mu_{0}^{\left(1\right)}$. For example, when $\mu_{0}^{\left(2\right)}=3\mu_{0}^{\left(1\right)}$, and the overall pre-compression is 10%, the compression strain in the softer layers is 12.3%, whereas that in the stiff layer is 5.5%. According to figure 13*b*, the hardening effect in the softer layers is more significant, indicating that the hardening effects within these layers are different. As shown in figure 13*c*, the axial strain ratio will drop to 1.7 instead of 3.0 when a pre-compression of 10% is applied (i.e. the contrast of the axial strain image will decrease). This issue has also been discussed by Shiina *et al*. [39] (figure 13*d*).

### (c) Mechanics of dynamic elastography with harmonic stimuli

For elastography methods with harmonic stimuli, the steady responses of the target tissues are typically used to determine the tissue mechanical properties. Then the inverse problem involved in this case may be divided into two categories: the modal analysis method and the shear wave analysis method.

Modal analysis methods include the vibration amplitude sonoelastography and SWIRE [55,61]. Taking a homogeneous medium with a hard/soft inclusion as an example, when the target soft material is forced to vibrate, a state-steady pattern that relies on the mechanical properties of the inclusion is formed. For example, a softer inclusion may yield larger local displacement. Thus, lesions within the tissues can be identified [56]. Moreover, when the frequency of the excitation reaches the resonance frequency of the soft inclusion, the amplitude of the displacement within the inclusion will reach the peak value. Therefore, the contrast between the inclusion and the surrounding tissue also increases. Under the condition that the inclusion is softer than the surrounding matrix, the uniqueness of the resonance mode can be guaranteed; therefore, the resonance frequency can also be used to quantitatively measure the mechanical properties of the inclusion, assisted by an FE model [61]. One challenge in modal analysis methods is that the modal shape relies strongly on the BCs [151]. For example, Gao *et al*. [55] used fixed BCs, whereas Schmitt *et al*. [61] used the so-called perfectly matched layer to avoid reflection of the shear waves [152]. Moreover, quantitatively inferring the material parameters from the experimental responses via modal analysis methods requires the use of the FEM, which might complicate the use of this technique by clinicians.

Most other DEHS methods, including vibration-phase gradient sonoelastography, CWI, HMI and SDUV, use the shear wave analysis method to extract the mechanical properties of soft tissues. The wave motion equation can be obtained by inserting equation (3.11) into equation (3.3)

3.36 $$\mu \nabla^{2}\text{u}\text{ + ( }\lambda +\mu \text{) }\nabla (\nabla \cdot \text{u})+\rho \text{b}=\rho \frac{\partial^{2}\text{u}}{\partial t^{2}}.$$

When considering the steady state of plane wave propagation, the body force *ρ***b** may be assumed to be zero.

To consider the dispersion of the shear wave resulting from the viscoelastic deformation of soft tissues, the viscosity of biological tissue must be considered by assuming that the stress depends on the derivatives of the strain components and the strain components themselves [153]. Thus, in equation (3.11), the Lamé constants should be written as

3.37 $$\lambda =\lambda_{1}+\lambda_{2}\frac{\partial ()}{\partial t}\;\text{and}\;\mu =\mu_{1}+\mu_{2}\frac{\partial ()}{\partial t},$$

where *μ*_2_ and *λ*_2_ are the coefficient of shear and the volume viscosity, respectively.

For the plane shear wave considered here, without loss of generality, we suppose that $u_{2}=u_{20}{\text{e}}^{i(kx_{1}-\omega t)}$ and that other displacement components are zero, where *ω* and *k* are the angular frequency and wavenumber, respectively. Thus, the transverse (shear) wave propagates along the *x*_1_-axis. Inserting *u*_2_ and equation (3.37) into equation (3.36), we obtain

3.38 $$k=\sqrt{\frac{\rho \omega^{2}}{\mu_{1}-\text{i}\omega \mu_{2}}},$$

where i denotes the imaginary unit. Because *k* is a complex number, its real part determines the phase velocity of the shear wave

3.39 $$c=\frac{\omega }{\text{Re}(k)},$$

whereas its imaginary part determines the attenuation of the wave [153,154]. Inserting equation (3.38) into equation (3.39), we obtain

3.40 $$c=\sqrt{\frac{2(\mu_{1}^{2}+\omega^{2}\mu_{2}^{2})}{\rho \left(\mu_{1}+\sqrt{\mu_{1}^{2}+\omega^{2}\mu_{2}^{2}}\right)}}.$$

The above equation has been used to describe the dispersion of low-frequency shear waves in biological tissues [16,29,36,74,75]. By fitting the experimental dispersion curve, both *μ*_1_ and *μ*_2_ can be obtained.

Based on shear wave elastography methods using harmonic stimuli, in theory, we can quantitatively measure both the elasticity and viscosity parameters of a homogeneous soft tissue. However, a drawback of this type of method is that the resolutions of the methods are limited by the low frequency of the shear waves which corresponds to a relatively large wavelength. When the typical dimension of a soft tissue (e.g. the size of a lesion) is comparable to or even smaller than the wavelength, the mechanical properties cannot be simply determined using an analytical solution such as the one given by equation (3.40).

### (d) Mechanics of dynamic elastography with transient stimuli

The mechanics of DETS is presented in this subsection. Considering that in many soft tissues are clearly anisotropic and that the effects of pre-stresses may come into play, elastography of anisotropic soft tissues and pre-deformed soft tissues is also discussed here. It should be pointed out that the mechanics models discussed here focus on the elastic medium. In this case, the plane wave assumption can be used to derive the correlation between the velocities of elastic waves and material parameters. However, if the medium is modelled as a viscoelastic material and the attenuation of the shear wave is used to infer the viscoelastic parameters, attenuation caused by both the geometry of the wavefront and the viscosity of the medium should be considered.

#### (i) Mechanical model for the transient elastography method

The mechanical model underlying the TE method may be simplified as the transient motion of an elastic half-space induced by a local vibrator imposed on the surface. The theoretical analysis of this issue dates back to the classical works of Lamb [155] and Pekeris [156]. The key results have been summarized in detail in the textbook [123] and are briefly presented here.

**Figure 14.**
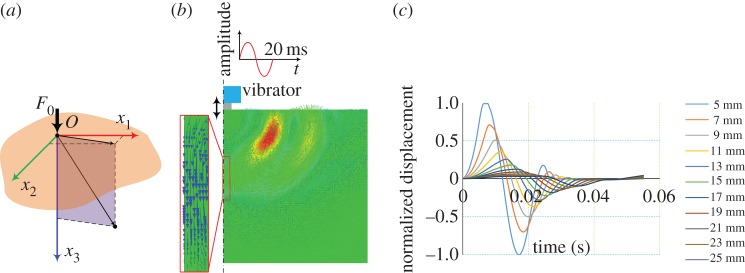
(*a*) Schematic of the Lamb problem (i.e. the motion of a half-space induced by a concentrated force). (*b*) Typical displacement field in the TE given by FEA. The frequency of the vibrator is 50 Hz and the loading process lasts 20 ms. (*c*) The axial displacements of the points on the axis at different depths (5 ∼ 25 mm). The displacements are normalized by the displacement at the depth of 5 mm. Based on the arrival times of the displacement peaks, the velocity of the transient wave can be determined. (Online version in colour.)

To consider the transient shear wave induced in TE, we first consider the motion of a half-space induced by a concentrated force perpendicular to the surface of the half-space, as shown in figure 14*a*. The BCs at *x*_3_ = 0 are

3.41 $$\sigma_{13}=0,\sigma_{23}=0\;\text{and}\;\sigma_{33}=F_{0}\delta (x_{1})\delta (x_{2}),$$

and the initial conditions (ICs) are

3.42 $$u_{i}=0\;\text{and}\;\;\frac{\partial u_{i}}{\partial t}=0,\;\text{when}\;t<0.$$

It is convenient to solve equation (3.36) with the above BCs and the ICs in cylindrical coordinates because this problem is axisymmetric. Achenbach solved this problem by using the integral transformation method [123]. Once this problem is solved, the displacement field in the TE induced by a local distribution force can be obtained using the superposition principle [151]. To demonstrate the key mechanics underlying the TE method, FE analysis (FEA) is performed here, and the results are shown in figure 14.

Figure 14*b* indicates the displacement field at a typical time revealed by FEA after the push by the vibrator. Clearly, the shear waves mainly propagate along the direction corresponding to an angle of approximately 45° relative to the vertical direction. The problem is axisymmetric; therefore, the displacements of the material points along the axis of symmetry (i.e. their polarized directions) are parallel to the loading direction. Because the propagation direction of the wave is also along the loading direction, the wave along the loading direction appears to be a longitudinal wave. By tracking the propagation velocity of this wave (i.e. the arrival time of the peak displacement at different points), as shown in figure 14*c*, the velocity of the wave is *c*_t_ instead of *c*_1_, where *c*_t_ and *c*_1_ are the bulk transverse (shear) and bulk longitudinal (compression) wave velocities, respectively, which are defined as [123]

3.43 $$c_{T}=\sqrt{\frac{\mu }{\rho }}\;\text{and}\;c_{L}=\sqrt{\frac{\lambda +2\mu }{\rho }}.$$

Sandrin *et al*. explained this phenomenon in terms of the diffraction effect [101]. In their recent work, Catheline & Benech [157] discussed this longitudinal shear wave in detail [157]. The longitudinal shear wave is shown to quickly decay when the wave propagates away; however, it can be monitored and tracked in TE because of its long wavelength in soft tissues.

By tracking the propagation of the longitudinal shear wave along the loading direction, the shear velocity *c*_T_ can be measured. Then, according to equation (3.43), the elastic modulus of the incompressible soft tissue can be determined as

3.44 $$E\approx 3\mu =3\rho c_{T}^{2},$$

assuming that the mass density of the soft biological tissue is known. Equation (3.44) is used in most transient shear wave elastography methods (e.g. TE, ARFI-based SWEI, SSI and CUSE) although these methods involve different mechanical models.

#### (ii) Mechanical model involved in ARFI-based shear wave elasticity imaging

Here, we consider the initial value problem involved in the ARFI-based SWEI method, which uses the focused ARF to induce shear waves in soft tissues. This issue may be modelled as the action of the focused ARF at an internal point in an infinite solid. The body force is determined by equation (2.1) and is given by

3.45 $$\text{f}=\delta (\text{x})\delta (t)\hat{\text{f}}.$$

Note that the amplitude of **f** in equation (3.45) can be arbitrary. Without loss of generality, $\hat{\text{f}}$ is parallel to the *x_m_*-axis (*m* = 1,2,3); therefore, $\hat{\text{f}}=\delta_{im}{\text{e}}_{i}$, where *δ_im_* is the Kronecker delta. Using the **f** given by equation (3.45) to replace *ρ***b** in equation (3.36), we can obtain the equilibrium equation in the following component form:

3.46 $$\mu u_{i,jj}\text{ + ( }\lambda +\mu \text{) }u_{j,ij}+\delta (\text{x})\delta (t)\delta_{im}=\rho \frac{\partial^{2}u_{i}}{\partial t^{2}}.$$

The above equation and the initial condition given by equation (3.42) form the initial value problem involved in SWEI. The solution to this problem is referred to as solution of the Green function [123,158,159], and that can be tracked with ultrasound elastography in soft tissues reads [34]

3.47 $$u_{im}^{\mathrm{Iso}}(\text{x},t)\approx \frac{1}{4\pi \rho c_{T}^{2}}(\delta_{im}-\gamma_{i}\gamma_{m})\frac{1}{r}\delta \left(t-\frac{r}{c_{T}}\right),$$

where *r* = |**x**|, $\gamma_{i}=x_{i}/r=\partial r/\partial x_{i}$. The superscript of $u_{im}^{\mathrm{Iso}}(\text{x},t)$ indicates that the solid is isotropic, and the subscript *m* indicates that **f** is applied along the *x_m_*-axis. Equation (3.47) indicates that the shear wave has a spherical wavefront in space. The propagation velocity of the wavefront is *c*_t_.

Note that according to the elastodynamic representation theorem [123], for any body force **f**(**x**,*t*) which may vary with time and coordinates, the solution to equation (3.36) can be obtained from the Green function solution by

3.48 $$u_{i}(\text{x},t)=\int \int f_{m}(\xi ,\tau )u_{im}^{\mathrm{Iso}}(\text{x}-\xi ,t-\tau )\;\text{d}\tau \;\text{d}\xi .$$

For example, various ultrasound push beams have been applied to induce shear waves within soft tissues in CUSE [96,112]. In this case, the distribution of the ARF may be diverse, and the resulting displacement field can be determined by solving equation (3.48) when the distribution of the body force is given.

#### (iii) Mechanics underlying supersonic shear imaging

In the SSI technique, the ultrasound beams are successively focused at different depths within the soft tissue (i.e. the ARF moves with a given velocity). In this case, the body force induced by the ultrasound beam can be simplified as

3.49 $$\text{f}=\delta (\text{x}-v_{f}t\hat{\text{f}})\hat{\text{f}},$$

where *v*_f_ is the moving velocity of the focused ARF. It should be noted that the ARF is discrete in a practical use and applied at different points instead of a continuous function as given in equation (3.49); however, such a simplification indeed can retain the physical nature of the problem and brings great ease for theoretical and computational modelling. Studying the elastodynamic fields produced by moving loads is a classical issue in mechanics, and many relevant works exist in the literature [160–162]. Here, the moving direction of the force is along $\hat{\text{f}}$. Without loss of generality, let $\hat{\text{f}}=(0,0,1)$, which indicates that the force is moving along *x*_3_.

We consider the case in which the moving velocity *v*_f_ is substantially greater than the velocity of the shear wave in the soft tissue. In practical applications of the SSI technique, the velocity of the moving ARF is essentially the velocity of the ultrasound wave, which is hundreds of times larger than the shear wave velocity in soft tissues. Inserting equations (3.49) and (3.47) into equation (3.48), the resulting displacement field induced by the moving force has been obtained as [34,107]

3.50 $$u_{i}(\text{x},t)=\frac{1}{\sqrt{1-M_{\mathrm{Iso}}^{2}\sin^{2}\Theta (t)}}F^{\mathrm{Iso}}(\text{x},t,\tau_{1}^{\mathrm{Iso}},\tau_{2}^{\mathrm{Iso}}),$$

where *M*_Iso_ = *v*_f_/*c*_t_ is defined as the Mach number,

3.51 $$\begin{array}{rl} & \tau_{1,2}^{\mathrm{Iso}}=t+\frac{R(t)}{c_{T}({M_{\mathrm{Iso}}}^{2}-1)}\left(M_{\mathrm{Iso}}\cos\Theta (t)\pm \sqrt{1-M_{\mathrm{Iso}}^{2}\sin^{2}\Theta (t)}\right), \end{array}$$

3.52 $$\begin{array}{rl} & F^{\mathrm{Iso}}(\text{x},t,\tau_{1}^{\mathrm{Iso}},\tau_{2}^{\mathrm{Iso}})=\sum_{k=1}^{2}\frac{1}{4\pi \mu R(t)}\left(\delta_{i3}-\frac{\partial R(\tau_{k}^{\mathrm{Iso}})}{\partial x_{i}}\frac{\partial R(\tau_{k}^{\mathrm{Iso}})}{\partial x_{3}}\right) \end{array}$$

3.53 $$\begin{array}{rl} \;\text{and}\; & \text{R}(t)=\text{x}-v_{f}t\text{a},\Theta (t)=\arccos\left(\frac{\text{R}\cdot \text{a}}{R}\right). \end{array}$$

The solution given by equation (3.50) forms the basis of the SSI technique [20,107]. According to equation (3.50), *M*_Iso_ > 1 confines the displacement within a circular cone (i.e. |sin*Θ*(*t*)| < 1/*M*_Iso_), indicating the formation of the shear-wave Mach cones. This phenomenon is the so-called ECE [107]. The Mach cone of the elastic wave within the elastic solid can also be induced by other stimuli, such as the movement of a dislocation. In this case, the formation of shear-wave Mach cones is also referred to as the elastodynamic Tamm problem, which has recently been studied by Lazar & Pellegrini [162]. In the SSI technique, the Mach number is large, and the angle of the Mach cone is very small. Therefore, quasi-plane waves form and the interfered wavefronts basically propagate in opposite directions. The moving velocity of interfered wavefronts can be measured to further determine the local elastic properties of soft tissues.

#### (iv) Mechanics of guided wave elastography

**Figure 15.**
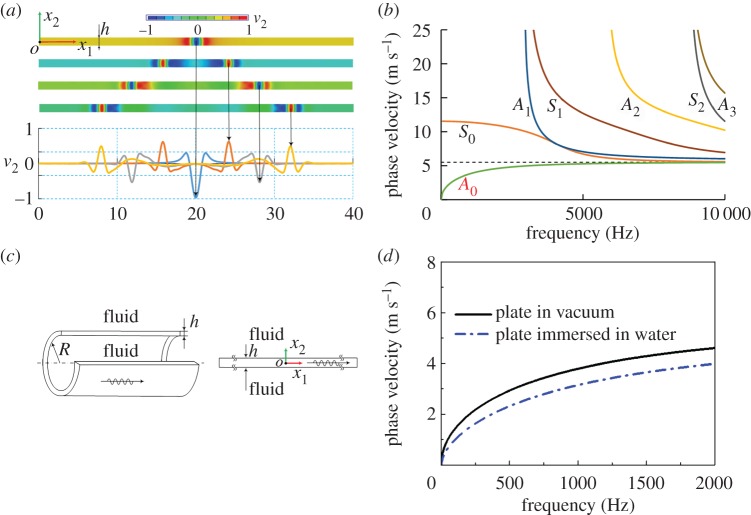
(*a*) Propagation of guided waves in an elastic plate. When the transient shear waves generated at the centre of the plate propagate in opposite directions, the wavefronts change constantly. (*b*) Dispersion relation of the antisymmetric and symmetric modes of the Lamb wave. (*c*) The simplified model used in GWE of the arterial wall. (*d*) The dispersion relations of the *A*_0_ mode in elastic plates in vacuum and immersed in water. (Online version in colour.)

The aforementioned methods (i.e. the TE, ARFI-based SWEI and SSI methods) use wave theories in infinite media to interpret experimental data and infer the material parameters by assuming that the wavelength of the shear wave is much smaller than the dimension of bulk tissues such as liver and breast. However, elastic waves in a thin-walled structure with thickness smaller than or comparable to the wavelength are guided. The guided waves are strongly dispersive as shown in figure 15*a*, and in this case, guided wave theory should be used. A typical guided wave is the so-called Lamb wave [123,124] (figure 15*a*). According to Lamb wave theory, only the wave modes of which the angular frequency *ω* and the wavenumber *k* satisfy the following dispersion equation:

3.54 $$\begin{array}{rl} {\left(k^{2}-q^{2}\right)}^{2}\tan\left(p\frac{h}{2}\right)+4k^{2}pq\tan\left(q\frac{h}{2}\right) & =0,\text{ for}\;\text{antisymmetric}\;\text{mode}\\ \;\text{and}\;{\left(k^{2}-q^{2}\right)}^{2}\cot\left(p\frac{h}{2}\right)+4k^{2}pq\cot\left(q\frac{h}{2}\right) & =0,\text{ for}\;\text{symmetric}\;\text{mode} \end{array}\}$$

can propagate within the elastic plate. In equation (3.54), $p=\sqrt{k_{l}^{2}-k^{2}}$, and $q=\sqrt{k_{t}^{2}-k^{2}}$, where *k*_1_ = *ω*/*c*_1_, and *k*_t_ = *ω*/*c*_t_. *h* denotes the thickness of the plate. Equation (3.54) can be solved numerically. As shown in figure 15*b*, both the antisymmetric and symmetric modes, denoted as *A_n_* and *S_n_* (*n* = 0, 1, 2,…), respectively, have countless branches. Guided waves such as Lamb waves have been widely used in non-destructive testing [163,164] and the mechanical characterization of engineering materials [165,166]. Unlike traditional engineering materials, elastic waves in soft materials (e.g. biological soft tissues) have much lower frequencies (typically no more than 2000 Hz). Therefore, only the dispersion curve of the zero-order antisymmetric mode (i.e. the *A*_0_ mode) is adopted in GWE methods.

GWE has promising applications in the characterization of arterial stiffness, which may potentially be used to diagnose some cardiovascular diseases [167,168]. Following previous studies [117,118], the arterial wall has been considered to be an elastic hollow cylinder surrounded by water both inside and outside. As shown in figure 15*c*, the inner radius and wall thickness of the hollow cylinder are *R* and *h*, respectively. Furthermore, the curvature of the hollow cylinder is ignored. This assumption will be discussed in detail later and is justified only when the frequency is sufficiently high. Then, the model can be simplified as an elastic plate immersed in fluid as shown in figure 15*c*. In this case, the dispersion equation is [169]

3.55 $$\begin{array}{rl} {\left(k^{2}-q^{2}\right)}^{2}\tan\left(p\frac{h}{2}\right)+4k^{2}pq\tan\left(q\frac{h}{2}\right)+\text{i}\frac{\rho_{F}p\omega^{4}}{\rho rc_{t}^{4}} & =0,\text{ for}\;\text{antisymmetric}\;\text{mode},\\ \;\text{and}\;{\left(k^{2}-q^{2}\right)}^{2}\cot\left(p\frac{h}{2}\right)+4k^{2}pq\cot\left(q\frac{h}{2}\right)-\text{i}\frac{\rho_{F}p\omega^{4}}{\rho rc_{t}^{4}} & =0,\text{ for}\;\text{symmetric}\;\text{mode, } \end{array}\}$$

where $r=\sqrt{\omega^{2}/c_{p}^{2}-k^{2}}$, $c_{p}=\sqrt{\kappa /\rho_{F}}$ is the velocity of pressure wave in the fluid, and *κ* and *ρ*_F_ are the bulk modulus and mass density of the fluid, respectively. Directly comparing equations (3.54) and (3.55) shows that the effect of the surrounding fluid appears in the third term of equation (3.55). When *ρ*_F_ is close to *ρ*, the surrounding fluid will significantly affect the dispersion relation, as shown in figure 15*d*. In practice, the fluid is considered to be water, for which *κ* = 2.2 GPa and *ρ*_F_ = 1000 kg m^–3^. The mass density of soft biological tissues is usually very close to that of water and typically assumed to be 1000 kg m^−3^. For a plate immersed in water, the phase velocities of the *A*_0_ mode are significantly smaller than those of a plate in vacuum within the frequency range of 0–2000 Hz. Equation (3.55) can be used to fit the experimental dispersion curve to infer the elastic modulus of the arterial wall.

Recently, vessel-mimicking phantom experiments have revealed that only the experimental dispersion curve in the high-frequency range can be well approximated by equation (3.55) [117,118]. Therefore, Maksuti *et al*. [170] adopted a frequency of 500 Hz as a critical frequency and used only the data beyond this critical frequency in the curve fitting. As mentioned above, this deviation stems from the assumption that the effect of curvature on the dispersion relation is negligible. In fact, this assumption is only valid when the frequency is sufficiently high [171]. The critical frequency, which is denoted as *f*_c_, and beyond which the Lamb wave model given by equation (3.55) works in principle, depends on both *R*/*h* and the elastic modulus of the cylinder. In a recent study, using dimensional analysis and systematic FE simulations, the explicit expression of the critical frequency was obtained [119]

3.56 $$f_{c}=0.304\frac{1}{h}\sqrt{\frac{E}{3\rho }}{\left(\frac{R}{h}\right)}^{-0.934}.$$

**Figure 16.**
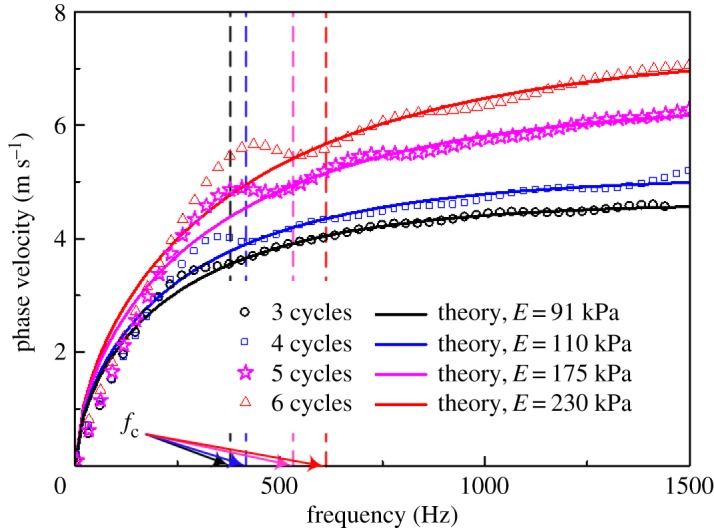
Experimental results (discrete points) obtained from vessel-mimicking phantoms with different elastic moduli are fitted with equation (3.55) (solid lines). The vessel-mimicking phantoms were made from polyvinyl alcohol cryogel which underwent different freezing/thawing cycles, i.e. three to six cycles, and had different elastic moduli. Both the critical frequencies and the elastic moduli of the phantoms are determined according to the method suggested in [119]. (Online version in colour.)

When the frequency exceeds *f*_c_, the relative error between the phase velocity given by equation (3.55) and that of the guided axial wave is less than 5%. Furthermore, an inverse approach based on equations (3.56) and (3.55) has been proposed to identify both the elastic modulus of an artery and the critical frequency from the experimental dispersion curve [119]. Typical results for vessel-mimicking phantoms with different elastic moduli are presented in figure 16.

#### (v) Shear wave elastography of anisotropic soft tissues

Many soft tissues such as skeletal muscles and tendons are anisotropic and may be described using the TI model given by equation (3.10). In the following discussion, the fibre direction is always along the *x*_3_-axis.

In anisotropic soft media, the velocities of shear waves are direction-dependent. The displacement caused by the plane wave is assumed to be $\text{u}=\text{U}{\text{e}}^{i(\text{k}\cdot \text{x}-\omega t)}$, where $\text{k}=k\hat{\text{k}}$ is the wavevector, *k* = |**k**| denotes the wavenumber, and *ω* represents the angular frequency. The phase velocity *c* is defined as *c* = *ω*/*k*, and **U **= *U_i_***e***_i_*, where **e***_i_* denotes the base vector. Inserting **u** into equation (3.10) to obtain the stress components, the equilibrium equation (3.3) can be written as

3.57 $$(k^{2}C_{ijkl}{\hat{k}}_{j}{\hat{k}}_{l}-\rho \omega^{2}\delta_{ik})U_{k}=0.$$

According to equation (3.57), the existence of a non-zero **U** requires

3.58 $$\det(k^{2}C_{ijkl}{\hat{k}}_{j}{\hat{k}}_{l}-\rho \omega^{2}\delta_{ik})=0.$$

From equation (3.58) and using the relationship between $C_{ijkl}$ and *μ*_T_, *μ*_L_ and *E*_L_, the two bulk shear wave speeds can be obtained from

3.59 $$\begin{array}{rl} \rho c_{\mathrm{SH}}^{2} & =\mu_{T}({\hat{k}}_{1}^{2}+{\hat{k}}_{2}^{2})+\mu_{L}{\hat{k}}_{3}^{2}\\ \;\text{and}\;\rho c_{\mathrm{qSV}}^{2} & =\mu_{L}+(E_{L}+\mu_{T}-4\mu_{L})(1-{\hat{k}}_{3}^{2}){\hat{k}}_{3}^{2}. \end{array}\}$$

**Figure 17.**
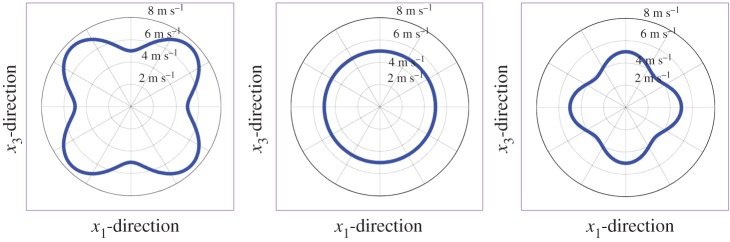
Plot of the phase velocity. Because *v*_qSV_ is axisymmetric, only the distribution of *v*_qSV_ in the *x*_1_–*x*_3_-plane is plotted. For all three cases, *μ*_T_ = 9 kPa, *μ*_L_ = 25 kPa, and the parameter *C* is (*a*) 62.5 kPa, (*b*) 0 and (*c*) −21.875 kPa, respectively. (Online version in colour.)

The subscripts ‘SH’ and ‘qSV’ denote the horizontally and quasi-vertically polarized shear waves, respectively [172]. Here, we define *C* = (*E*_L_ + *μ*_T_−4*μ*_L_)/2. Note *C* = 0 denotes a special type of TI material; in this case, the phase velocity of the ‘qSV’ mode becomes isotropic, as shown in figure 17. Besides, the group velocity, which is defined as **c**_g_ = ∂*ω*/∂**k** (i.e. the velocity of a wave packet) can be obtained according to equation (3.58).

**Figure 18.**
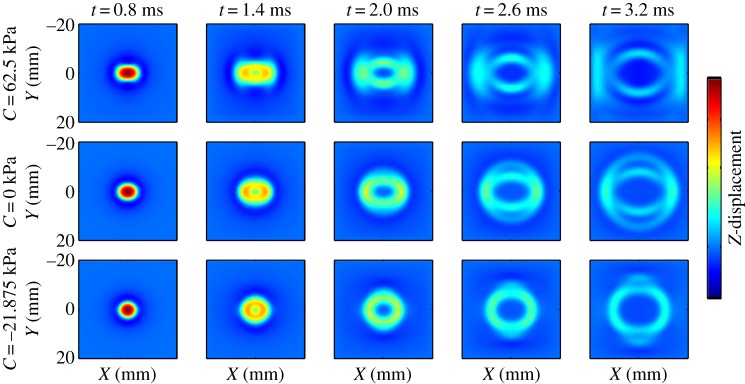
FE simulation of shear wave propagation in TI materials with different values of *C*. For all three cases, *μ*_T_ = 9 kPa, and *μ*_L_ = 25 kPa. The direction of the focused ARF is applied along the *Z*-axis, which has a 45° angle with the *x*_3_-axis (fibre direction) [35]. Reprinted from reference [35] with permission. (Online version in colour.)

When measuring the arrival time of the shear waves generated by a concentrated force, the group velocity can be determined from the travelling distance of the wave divided by the arrival time [154,173]. In SWEI, the group velocities can be measured in this way, and the mechanical parameters can be determined [35,106]. Figure 18 shows the shear waves in TI media generated by a simulated ARF [35]. The force is applied along the *Z*-direction, which has an angle of 45° with the *x*_3_-axis direction (fibre direction); therefore, both shear wave modes are induced. The wavefront propagates away from the load point at the group velocity. Clearly, for materials with different values of *C*, the shapes of wavefronts for the qSV mode are different, as predicted by equation (3.59). Because the qSV mode, which is related to *E*_L_, can be induced in this way, Rouze *et al*. [35] suggest using the ARFI-based SWEI with the experimental set-up described by their FEA to fully characterize the elastic parameters of a TI material.

**Figure 19.**
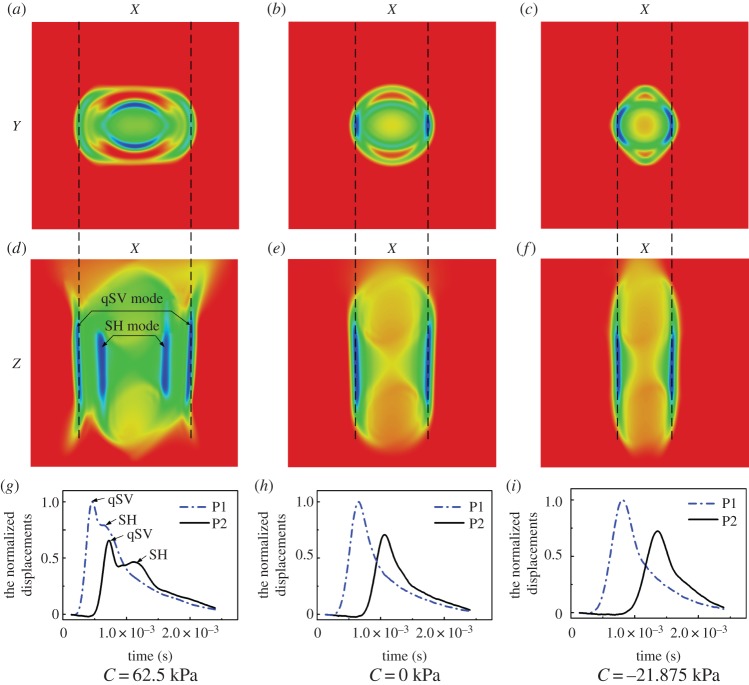
The ECE at a high Mach number when the angle between the direction of the moving force (*Z*-axis) and the fibre direction (*x*_3_-axis) is taken as 45°. (*a*,*d*,*g*) *C* > 0; (*b*,*e*,*h*) *C* = 0; (*c*,*f*,*i*) *C* < 0. For all three cases *μ*_T_ = 9 kPa, and *μ*_L_ = 25 kPa [34]. (Online version in colour.)

More conveniently, if the phase velocities can be measured, then equation (3.59) can be used to determine the mechanical properties. The phase velocities are easy to measure for the plane waves. Because of the ECE in the isotropic elastic medium, quasi-plane waves can be generated within the soft tissue by the ARF moving at a high speed. Addressing the ECE in the anisotropic elastic media is very important for the determination of the anisotropic elastic parameters of soft tissues. Li *et al*. [34] recently studied and revealed the ECE in TI medium through both theoretical analysis and numerical simulations. Figure 19*a*–*f* shows the displacement field when the moving direction of the ARF has a 45° angle with the fibre direction. According to equation (3.59), the phase velocity of the SH mode is only dependent on the two shear moduli *μ*_T_ and *μ*_L_ [36,174]. However, the phase velocity qSV mode is highly dependent on parameter *C* (or *E*_L_). For illustrative purposes, the normalized displacements of the qSV mode at two points located on the *X*-axis (denoted as P_1_ and P_2_ respectively) are plotted in figure 19*g*–*i*. The shear wave velocity can be determined from the arrival time of the peak displacement. In this case, the phase velocities of the qSV mode with wavevector $\hat{\text{k}}=\left(\sqrt{2}/2,0,\sqrt{2}/2\right)$ can be determined [34]. Then, according to equation (3.59), we have

3.60 $$E_{L}=4\rho c_{{\mathrm{qSV},45}^{\circ }}^{2}-\mu_{T},$$

where $c_{{\mathrm{qSV},45}^{\circ }}^{}$ denotes the velocity of the interfered wavefronts measured from figure 19*g*–*i*.

**Figure 20.**
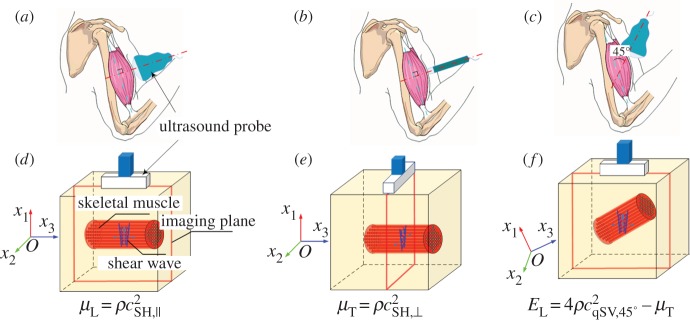
The SSI-based experimental protocol used to fully characterize skeletal muscles. (*a,d*) The SH mode shear wave is generated along the fibre direction and its velocity, $c_{\mathrm{SH},∥}$, is measured. According to equation (3.59), *μ*_L_ can be measured as $\mu_{L}=\rho c_{\mathrm{SH},∥}^{2}$. (*b,e*) Similarly, *μ*_T_ can be measured using the SH mode shear wave propagating perpendicular to the fibre according to $\mu_{T}=\rho c_{\mathrm{SH},⊥}^{2}$. (*c,f*) In this step, the velocity of the qSV mode shear wave, $c_{{\mathrm{qSV},45}^{\circ }}^{}$, is measured. Then *E*_L_ can be determined as $E_{L}=4\rho c_{{\mathrm{qSV},45}^{\circ }}^{2}-\mu_{T}$ [33]. (Online version in colour.)

To fully determine the mechanical properties of the TI medium, the experimental set-up shown in figure 20 has been proposed [33]. When the ultrasound probe is placed as shown in figure 20, the qSV mode shear wave can be generated and used to measure *E*_L_ if *μ*_T_ has been determined. Such an experimental protocol can be easily realized using the SSI technique, and the three independent elastic parameters of skeletal muscles can be measured *in vivo*. Experiments have been conducted on the biceps brachii and gastrocnemius muscles of volunteers [33], and the shear moduli determined are in good agreement with previously reported data [36,47,175–177].

#### (vi) Shear wave elastography of pre-stressed soft tissues

In most ultrasound elastography measurements, the soft tissue it is assumed to be stress free. However, in practical measurements, the contact between the probe and soft tissues may lead to finite deformation. Moreover, analysing the propagation of the shear wave in a pre-stressed soft tissue enables the determination of the tissue's hyperelastic parameters. The effect of pre-stress on wave propagation has been investigated previously [26,178–181]. Here, we use the incremental dynamic theory described in §3a to address the effect of pre-stress on the propagation velocities of shear waves in soft tissues [8,34,131,133,134,182].

The deformation gradient (i.e. **F**) induced by the pre-deformation is assumed to be homogeneous in the ROIs. In this case, equation (3.29) can be simplified as

3.61 $$A_{0piqj}u_{j,pq}-{\dot{p}}_{,i}-\rho \frac{\partial^{2}{\dot{u}}_{i}}{\partial t^{2}}=0.$$

**Figure 21.**
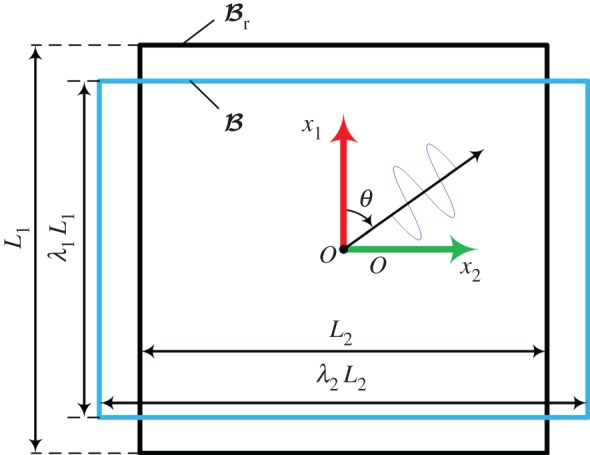
Schematic of the finite deformation of the tested material and the wave propagation. (Online version in colour.)

According to equation (3.22), $\text{tr(}\partial \dot{\text{u}}/\partial \text{x}\text{) = }{\dot{u}}_{i,i}=0$ by the incompressibility constraint. Furthermore, the deformation gradient can be written as **F **= diag(*λ*_1_,*λ*_2_,*λ*_3_), where *λ_i_* (*i* = 1,2,3) denotes the stretch ratio along the *x_i_*-axis (figure 21), and *λ*_1_*λ*_2_*λ*_3_ = 1 because of the incompressibility constraint. Under these conditions, the fourth-order tensor $A_{0piqj}$ can be determined according to equation (3.28)

First, we consider isotropic hyperelastic materials. For a plane wave propagating within the plane of *x*_1_ − *x*_2_, i.e. ${\dot{u}}_{3}=0$, and we can define, ${\dot{u}}_{1}=\psi_{,2}$, and ${\dot{u}}_{2}=-\psi_{,1}$. *ψ* is a scalar function of (*x*_1_,*x*_2_,*t*) and is assumed in the form

3.62 $$\psi =\psi_{0}\exp[\text{i}k(x_{1}\cos\theta +x_{2}\sin\theta -ct)],$$

where *k* and *c* denote the wavenumber and phase velocity, respectively, and *θ* denotes the wave propagation direction, as shown in figure 21. Inserting the incremental displacement components into equation (3.61) and eliminating $\dot{p}$, we can obtain the explicit expression for the phase velocity [133,139]

3.63 $$(C_{1}+C_{3}-2C_{2})\cos^{4}\theta +2(C_{2}-C_{3})\cos^{2}\theta +C_{3}=\rho c^{2},$$

where

3.64 $$C_{1}=A_{01212},2C_{2}=A_{01111}+A_{02222}-2A_{01122}-2A_{01221},\;C_{3}=A_{02121}.$$

According to equation (3.63), the propagation velocity is direction-dependent and depends on the choice of constitutive laws. For example, when the Demiray–Fung model is adopted and *θ* = 90°, we obtain

3.65 $$\rho c^{2}=\mu_{0}\lambda_{2}^{2}{\text{e}}^{b(\lambda_{1}^{2}+\lambda_{2}^{2}+\lambda_{3}^{2}-3)}.$$

In practice, we may compress the soft tissue along the *x*_1_-axis, i.e. *λ*_1_ = *λ* is prescribed. Then we define *λ*_2_ = *λ^−ξ^*, where the parameter *ξ* is determined by the deformation state within the soft solid; for example, *ξ* = 0.5 indicates uniaxial compression, whereas *ξ* = 1 represents the plane-stain state. In practical measurements, *ξ* can be measured [8,27]. Using those notations, equation (3.65) can be written as

3.66 $$\rho c^{2}=\mu_{0}\lambda_{}^{-2\xi }{\text{e}}^{b(\lambda^{2}+\lambda_{}^{-2\xi }+\lambda_{}^{-2(1-\xi )}-3)}.$$

**Figure 22.**
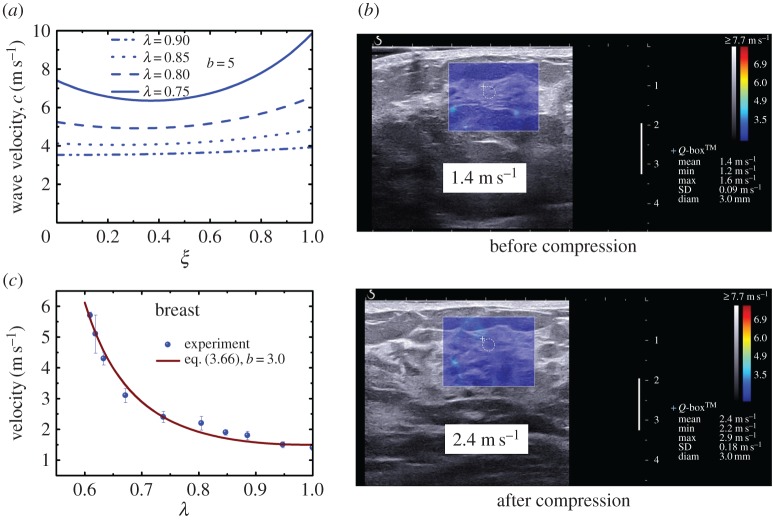
(*a*) Dependence of the wave speed on parameter *ξ* for different *λ*. (*b*) The velocities of the shear waves in breast tissues before and after compression. (*c*) Variation of the shear wave velocities with compression strains determined by SSI, which can be fitted with equation (3.66) to obtain *b* [8]. (Online version in colour.)

Figure 22*a* shows the dependence of the wave speed on parameter *ξ* for different *λ* when *b* = 5. Clearly, when 0.1 ≤ *ξ* ≤ 0.8, the effect of *ξ* on the wave velocity is not significant. Equation (3.66) provides the relationship between the pre-deformation and the shear wave velocities, from which the effect of pre-deformation on the wave propagation can be quantitatively evaluated. Moreover, based on equation (3.66), Jiang *et al*. established an inverse approach and measured the hardening parameter *b* of human breast and human heel fat pad tissues *in vivo* and porcine brains *ex vivo* [8,27]. Figure 22*b*,*c* shows the shear wave velocities measured before and after compression. By fitting the shear wave velocities at different compression strains with equation (3.66), the hardening parameter *b* can be obtained.

**Figure 23.**
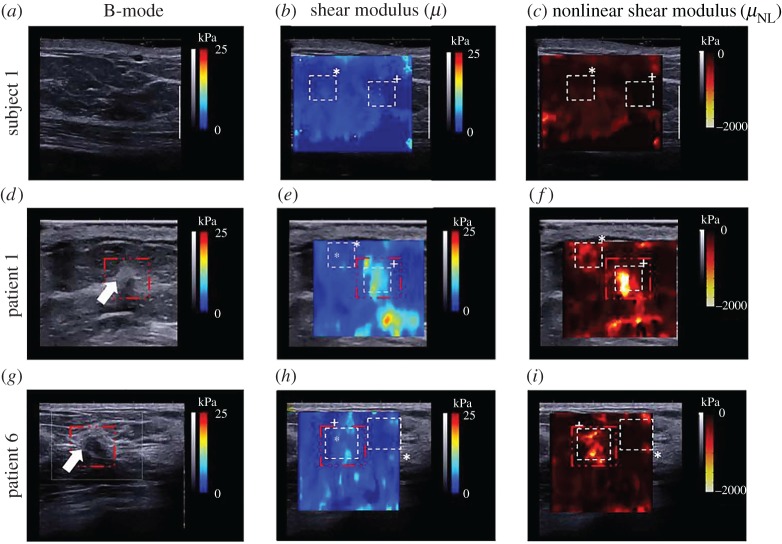
Experimental results obtained for three patients: a healthy volunteer, a patient with a tumour and a patient with a benign lesion. (*a,d,g*) B-mode images, (*b,e,h*) shear moduli and (*c,f,i*) nonlinear parameter *A* values [28]. Reprinted from reference [28] with permission. (Online version in colour.)

Effects of the pre-stress on wave propagation have been studied [26–28,182] for isotropic hyperelastic materials described with the constitutive law given by equation (3.15). The following analytical solution was derived:

3.67 $$\rho c^{2}=\mu_{0}-\frac{A}{12\mu_{0}}\sigma_{11},$$

assuming that the soft material is loaded with uniaxial pre-compression stress *σ*_11_ along *x*_1_-axis and that the shear wave is propagating along the *x*_2_-axis. Using equation (3.67), parameter *A* has been measured for agar-gelatine and polyvinyl alcohol cryogel phantoms. In their recent work, Bernal *et al*. [28] further adopted equation (3.67) to establish an inverse approach to measure *A*. In their approach, the strain field is measured using static elastography, and, thus, the stress field can be determined. By gradually increasing the compression and measuring the incremental strain and shear wave velocities, the relationship between the stress and the shear wave velocity can be obtained and used to determine *A* according to equation (3.67). The initial experiments, as shown in figure 23, have demonstrated the potential of this method for enhancing the contrast between a lesion and its surrounding tissues [28].

In addition to isotropic hyperelastic materials, the effects of pre-stress on shear wave propagation in TI hyperelastic materials have also been studied [33,34,183–185]. For the constitutive law given by equation (3.21), the phase velocity of the SH mode shear wave can be obtained as

**Figure 24.**
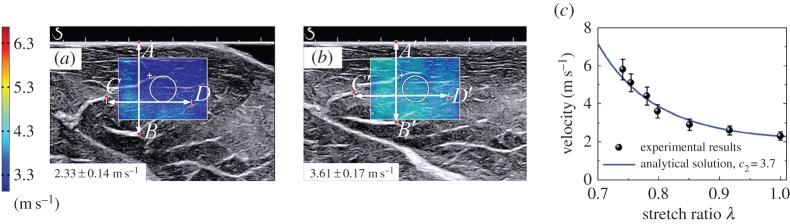
The shear wave velocities of the beef *ex vivo* (*a*) before and (*b*) after compression. (*c*) By fitting the variation of the shear wave velocities with the compression strain, the parameter *c*_2_ was determined [33]. (Online version in colour.)

3.68 ρc2=μTec2(I1−3)λ12sin2θ+[μTec2(I1−3)+2(μT−μL)+(EL+μT−4μL)ec4(λ3−1)2(1−1λ3)− (μT−μL)(2λ32+λ22)(1−1λ3)]λ32cos2θ,

where *I*_1_ is defined in equation (3.13). When no pre-deformation exists (i.e. *λ*_1_ = *λ*_2_ = *λ*_3_ = 1), equation (3.68) reduces to equation (3.59). The hardening parameter *c*_2_ can be measured by using the inverse method proposed in [34]. The experiments conducted in [33] determined the hardening parameter *c*_2_ of beef muscles *ex vivo* (figure 24).

## 4. Discussion

**Figure 25.**
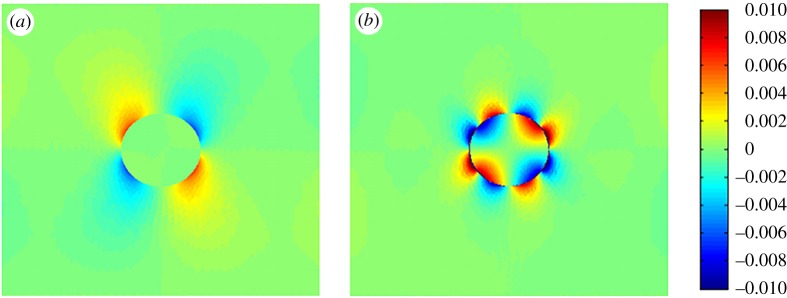
Axial-shear strain images from FEM of (*a*) a firmly bonded inclusion and (*b*) a loosely bonded inclusion. The coefficient of friction was 0.01, and the applied axial strain was 2%. The inclusion was twice stiffer than the background in both cases [186]. Reprinted from reference [186] with permission. (Online version in colour.)

This paper provides an overview of both static elastography and dynamic elastography with focus on the mechanics principles underlying these methods. Static elastography is relatively easy to realize and can qualitatively differentiate between soft tissues with different stiffnesses because soft tissues with different elastic moduli will undergo different amounts of deformation under external or internal stimuli. In addition to the compression strain along the loading direction, other deformation information may be acquired in static elastography. For instance, the interfacial bonding conditions between a benign tumour and the surrounding soft tissue and those between a malignant tumour and surrounding soft tissue are usually different. Therefore, by tracking the deformation at the interface (e.g. the shear strains at the interface), it is possible to differentiate a malignant tumour from a benign one using the static elastography method [186], as illustrated in figure 25. Here, the challenge is to accurately evaluate shear strains at the interface, which deserves further efforts.

In DEHS, a vibration source with a given frequency can be used to stimulate the soft tissue. Low-frequency (typically 100 Hz) shear waves generated in the soft tissue in the steady state are relatively easy to track using ultrasound imaging methods. However, a low-frequency shear wave corresponds to a relatively large wavelength. When the typical dimension of a soft tissue (e.g. the size of a lesion or inclusion) is comparable to or even smaller than the wavelength, its mechanical properties cannot be simply determined using an analytical solution such as equation (3.40). By contrast, shear waves generated in DETS are generally broad-band, and include more information that can be used to infer the material parameters. The centre frequency is typically 1000 Hz; therefore, the DETS in theory has better resolution than DEHS. In this sense, the DETS methods may be more suitable for evaluating the mechanical properties of soft tissues with finite dimensions. For instance, for a multilayer system, e.g. human skin [187], when a harmonic vibrator is used to induce shear waves in the dermis layer, the shear wave velocity depends not only on the material parameters of the dermis layer, but also on the mechanical properties of the adjacent layers and the geometrical parameters of the composites [187]. However, FEA shows that when a DETS method (e.g. the SSI technique) is used in this case, the velocity of the interfered wavefronts mainly depends on the mechanical properties of the dermis layer, and the effects of other parameters in the system are rather weak [187]. However, it should be noted that the viscosity of soft biological tissues may strongly attenuate the high-frequency shear waves and can significantly reduce the signal-to-noise ratio during the propagation of transient waves and affects the accuracy of wave velocities measured in a DETS method.

Although previous studies have demonstrated the usefulness of dynamic elastography methods for practical measurements, quantitatively measuring tissue mechanical properties using these methods remains challenging in many cases. Here, we take the GWE of arteries as an example. Real arterial walls have layered structures and GWE methods described in the literature are only able to determine the effective elastic modulus of the arterial wall [122]. Second, real artery tissues are anisotropic and contain distributed collagen fibres [142]. Although recent studies have demonstrated that determining the anisotropic and hyperelastic properties of a bulk anisotropic soft tissue is possible [33,34], assessing the anisotropic parameters of arterial walls using the shear wave elastography method is by no means trivial because the wave in the arterial wall is guided. Third, the modelled arterial wall is usually assumed to be a time-independent material, whereas a real artery may exhibit viscoelastic deformation. For viscoelastic soft tissues, the wavenumber *k* is no longer a real number but could be a complex number, reflecting the dissipation of the wave. Chan & Cawley [188] studied the dispersion curve of a viscoelastic plate. Their results showed that the lowest mode (i.e. the mode adopted in the inverse analysis) was essentially unaffected by the viscosity of the soft media. They also demonstrated that the high-order modes suffered from viscoelastic effects. Therefore, caution should be taken when adopting these higher-order modes. Fourth, in the literature, the outer region of the arterial wall was simplified to a non-viscous fluid to derive the theoretical solution in recent GWE methods. This assumption is only reasonable in some cases, such as carotid arteries, in which the elastic moduli of the arterial walls can be much greater than those of the perivascular tissues. Fifth, the effects of blood pressure in *in vivo* experiments cannot be ignored. In this case, a robust GWE method should consider guided waves in a pre-stressed soft tube.

Finally, note that identifying the material parameters using either static elastography or dynamic elastography represents an inverse problem. As mentioned above, an inverse problem may suffer from the issues of solution existence, uniqueness and stability. Among these issues, the stability of the solution is usually the key because the lack of stability will make the solution of an inverse problem have nothing to do with the real solution. Recently, Jiang *et al*. [8] introduced the concept of the condition number in analysing the stability of nonlinear elastic parameter *A* determined using the SSI technique via acoustoelasticity theory. For the strain energy density function given by equation (3.15), the following condition number in closed-form was derived in their study

4.1 $$\Psi =\frac{c}{A}\frac{\Delta A}{\Delta c}=2+\frac{2\Gamma_{0}\mu_{0}}{\Gamma_{1}A},$$

where $\Gamma_{0}=(\lambda^{2-2\xi }+\lambda^{-4\xi }-\lambda^{-2\xi })$, $\Gamma_{1}=(-5\lambda^{-2\xi }/4+\lambda^{-2}/4+(\lambda^{2-2\xi }+\lambda^{-4\xi })/2)$, and Δ*c* denotes the measurement error of the shear wave velocity *c* in experiments, which leads to an error Δ*A* in the identified *A*. Here *λ* and *ξ* are parameters describing the deformation of a soft tissue in the ROI [8]. Accordingly, the *condition number* measures the sensitivity of the identified solution of an inverse problem to data errors. The larger the condition number is, the more sensitive the identified solution to data errors will be. For example, when the condition number is 5, an error of 5% in the input data will lead to an error of 25% in the identified solution. Equation (4.1) reveals that sufficient compression (e.g. *λ* < 0.75) should be imposed on the soft tissue to decrease the condition number.

Therefore, to develop a robust elastography method for characterizing the mechanical properties of soft tissues or other soft materials, the existence, unique and stability issues of the solution to the inverse problem must be addressed. However, this issue has not received sufficient attention in the literature regarding ultrasound elastography.

## 5. Concluding remarks

Ultrasound-based elastography has emerged as a highly useful technique for characterizing the mechanical properties of soft materials, including living soft tissues, because of the extensive experimental and theoretical research performed in recent years, which has improved understanding of this technique and its applications, particularly in clinics. From the viewpoint of continuum mechanics, recent findings have contributed to shaping a set of unanswered questions that require investigations in future studies of static and dynamic elastography methods. Researchers, particularly those in the mechanics community, deserve to pay attention to these important issues. Some of these questions include the following: (i) How can we improve the current elastography methods based on the knowledge from the field of continuum mechanics to quantitatively determine the mechanical properties of soft tissues in critical cases, such as when the soft tissues are anisotropic and/or have finite dimensions (e.g. tumours) that significantly influence the propagation of shear waves? (ii) What opportunities exist for developing robust GWE methods to characterize the mechanical properties of thin-walled soft tissues, including arteries and bladder, *in vivo* based on the knowledge of guided wave in pre-stressed thin-walled soft solids? (iii) Diseases may alter the structures and functions of soft tissues/organs and change their mechanical properties. How can we model the diseased tissues/organs and determine which mechanical parameters (e.g. elastic, hyperelastic, viscoelastic and poroelastic parameters) are sensitive to the diseases, and further inspire the development of new elastography techniques? (iv) What new fundamental science can be explored (e.g. the propagation of elastic waves induced by a moving vibration source in pre-stressed inhomogeneous living soft tissues across different length scales)? (v) What new techniques are becoming available that could expand applications of ultrasound elastography and provide new opportunities to characterize diverse soft materials far beyond biological soft tissues?

Although the practical use of ultrasound elastography in quantitatively measurements of material parameters still faces challenges in many cases, based on the advances made in understanding this promising technique in recent years and the aforementioned opportunities for further study, one can reasonably predict that this technique has a bright future in a variety of fields, including not only medicine, but also biology, materials science, tissue engineering and soft matter physics. Note that this review gives emphasis to the mechanics theories involved in ultrasound elastography. Although understanding the responses of soft materials to various internal or external stimuli is by no means trivial, the knowledge obtained from continuum mechanics indeed helps yield some analytical solutions that can be used to interpret the experimental data.

Finally, we conclude this review with an old aphorism: ‘Simplicity is beauty’. Aleksandr Solzhenitsyn said, in his 1970 Nobel Prize speech, that ‘Beauty will save the world’. By pursuing fundamental solutions in simple forms that reveal the correlation between experimental responses and material parameters within the framework of continuum mechanics, we are not merely pursuing ‘beauty’ but also providing fundamental solutions that contribute to understanding ultrasound elastography methods and facilitate their practical use, particularly in medicine.
